# Rapid growth in a large Cambrian apex predator

**DOI:** 10.1093/nsr/nwad284

**Published:** 2023-11-03

**Authors:** Yu Wu, Stephen Pates, Daniel Pauly, Xingliang Zhang, Dongjing Fu

**Affiliations:** State Key Laboratory of Continental Dynamics and Shaanxi Key Laboratory of Early Life and Environment, Department of Geology, Northwest University, Xi’an 710069, China; Department of Zoology, University of Cambridge, Cambridge CB2 3EJ, UK; Department of Zoology, University of Cambridge, Cambridge CB2 3EJ, UK; Institute for the Oceans and Fisheries, University of British Columbia, Vancouver, BC V6T 1Z4, Canada; State Key Laboratory of Continental Dynamics and Shaanxi Key Laboratory of Early Life and Environment, Department of Geology, Northwest University, Xi’an 710069, China; Nanjing Institute of Geology and Palaeontology, Chinese Academy of Sciences, Nanjing 210008, China; State Key Laboratory of Continental Dynamics and Shaanxi Key Laboratory of Early Life and Environment, Department of Geology, Northwest University, Xi’an 710069, China

**Keywords:** stem-group Euarthropoda, Radiodonta, post-embryonic development, growth dynamics, Cambrian Explosion

## Abstract

Despite the importance of ontogenetic data on early diverging euarthropods to our understanding of the ecology and evolution of past life, the data are distinctly lacking, as reconstructing life histories of fossil animals is often challenging. Here we report the growth trajectory of frontal appendages of the apex predator *Amplectobelua symbrachiata*, one of the most common radiodont arthropods from the early Cambrian Chengjiang biota (*c*. 520 Ma) of China. Analysis of 432 specimens (9.1–137.1 mm length; 1.3–25.6 mm height) reveals that appendages grew isometrically, with an estimated maximum size of the whole animal of *c*. 90 cm. Individuals grew rapidly compared to extant arthropods, as assessed using the electronic length-frequency analysis (ELEFAN) approach. Therefore, we show that the Cambrian apex predator *A. symbrachiata* was an extremely fast-growing arthropod, with an unusual life history strategy that formed as part of the escalatory ‘arms race’ that shaped the Cambrian explosion over 500 Ma.

## Teaser

The post-embryonic development of a 520 million years ago apex predator was quantitively described, which revealed that Amplectobelua symbrachiata was an extremely fast-growing arthropod with an unusual life history strategy.

## INTRODUCTION

Exceptional Palaeozoic deposits preserving soft-bodied and lightly sclerotized material, such as the famous Chengjiang biota, Burgess Shale and more recently discovered Qingjiang biota, continue to provide insights into the early evolution of the Euarthropoda [1,2]—a diverse phylum that includes chelicerates, myriapods and pancrustaceans including insects—and the makeup of early animal ecosystems [3,4].

Studies on the post-embryonic development of Cambrian euarthropods are of high value for our understanding of the evolution of Euarthropoda, as evolutionary pressures act on all stages of development. Insights into the development of early euarthropods are also important for furthering our understanding of the ecology of Cambrian ecosystems, as juvenile euarthropods can occupy different niches to adults of the same species [5,6]. Such studies have been conducted on a range of upper stem-group and crown-group euarthropods, including fuxianhuiids [7], hymenocarines [8], isoxyids [9,10], megacheirans [5,11] and trilobites [12–14]. Our knowledge of ontogeny within the lower stem-group of Euarthropoda (*sensu* [15]), which charts the evolution from a lobopodian-like ancestor to the earliest diverging euarthropod group with arthropodized appendages, Radiodonta (*Amplectobelua, Anomalocaris, Hurdia* and relatives) [2,15,16], is more limited. To date, segment addition and allometry of some morphological features has been reported from a hurdiid radiodont *Stanleycaris* [17], and adult like morphology of a juvenile has been demonstrated for the amplectobeluid radiodont *Lyrarapax* [18]. Beyond these studies of complete bodies, miniature individual appendages [19] and carapaces [20] have been interpreted as juveniles, lens size changes and addition have been documented [21], and smaller and larger frontal appendages of individual species have been qualitatively compared [18].

Knowledge of growth ratios between instars, moulting frequencies and mortality estimates have remained unexplored for radiodonts and lower stem-group euarthropods more broadly. These factors are of great significance as they can greatly influence larger scale evolutionary trends [22–24]. Indeed, the act of moulting represents one of the most vulnerable periods in the life cycle of euarthropods [25], and the Investment Principle suggests that ecological trade-offs lead to very different numbers of ecdysis events and growth ratios in ecdysozoans [26].

**Figure 1.**
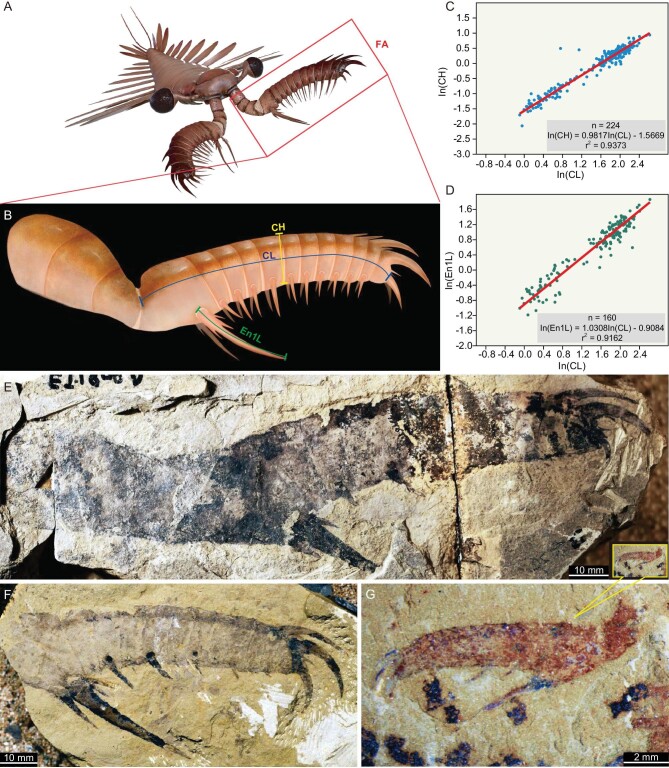
Adult and juvenile specimens of an *Amplectobelua symbrachiata* frontal appendage and statistical analysis for the ontogenetic relationship between main appendage traits. (A) General morphology of *A. symbrachiata*. (B) Morphology of frontal appendage (FA) and explanations of measurements taken for main analysis: claw length (CL), claw height (CH) and En1 length (En1L). (C and D) Scatter plots of the ontogenetic relationship between the independent variables of CL, CH and En1L, showing that CL, CH and En1L increase in size proportionally. n, number of specimens. (C) In(CL) regressed against In(CH). (D) In(CL) regressed against In(En1L). (E–G) Specimens of frontal appendages of different sizes. (E) The largest appendage (EJ-1908) and the smallest juvenile appendage (JS-0850), emphasizing the great size difference. (F) An adult appendage of medium size (SJZ-281). (G) Enlarged view of the smallest appendage (JS-0850). Measurements in cm. Source data provided in Supplementary Dataset 1.

Here we describe the post-embryonic development of frontal appendages in *Amplectobelua symbrachiata* (Fig. 1A), the most common radiodont from the early Cambrian (Series 2 Stage 3, ∼518 Ma) Chengjiang biota in South China (for more detail see Supplementary Note 1), using the expectation-maximization (EM) algorithm (for more details see Supplementary Notes 1 and 2) and ELEFAN (electronic length-frequency analysis; for more details see Supplementary Notes 6 and 7). These demonstrate that length/frequency data of *A. symbrachiata* appendages form a multimodal distribution, and provide estimates of absolute growth and mortality parameters for a radiodont for the first time. ELEFAN was initially developed to study the growth of tropical fish and of arthropods such as penaeid shrimps [27–29]. Widely used in fishery science and marine biology, it was recently applied in palaeontology, to an Ordovician trilobite [30]. Jointly, the two approaches allowed the most comprehensive characterization of the ontogeny of a lower stem-group euarthropod to date, and provide quantitative information on the growth trajectory of a radiodont for the first time.

## RESULTS

### Isometric growth of the whole appendage and individual podomeres

Measurements of claw length (CL) ranged from 9.1 mm to 137.1 mm (mean 53.5 mm), and claw height (CH) from 1.3 mm to 25.6 mm (mean 10.8 mm) (Fig. 1B; Supplementary Tables S1 and S2; Supplementary Datasets 1–3). The reduced major axis (RMA) regression analysis on the natural log of the linear morphometric measurements demonstrates that height grows proportionally to length for the claw of *A. symbrachiata* appendages—they display isometric growth (confidence level = 95%; In(CH) = 0.9817In(CL)–1.5669, r^2^ = 0.9373) (Fig. 1C; Supplementary Table S3). Individual claw podomeres display proportional growth between podomere height and length (Supplementary Fig. S1 and Supplementary Table S3), confirming the qualitative assessment that juvenile frontal appendages of *A. symbrachiata* have adult-like morphologies. The exceptionally long endite on the proximal-most claw podomere (En1) is a diagnostic feature of *A. symbrachiata* and other amolectobeluid radiodonts [18,31], more broadly. The length of En1 (En1L) ranged from 3.1 mm to 64.5 mm, and RMA indicated that En1 also grew proportional to CL (In(En1L) = 1.0308In(CL)–0.9084, r^2^ = 0.9162) (Fig. 1D).

### Recognition of overlapping normal distributions

Visual inspection of histograms suggested size/frequency data (CH and CL of frontal appendages) were composed of overlapping normal distributions. An EM algorithm recovered three overlapping normal distributions from these data (Supplementary Fig. S2; for further details see Supplementary Note 2). The mean size of each distribution was divided by the mean size at the previous distribution, giving a ratio of 3.3–3.4 (group 2 from group 1) and 1.5–1.7 (group 3 from group 2) (Supplementary Note 2). A *c*. 3 : 1 ratio between the means of the first two distributions is also recovered if EM is used to separate the data into two, four or five distributions.

### Comparison of growth ratios across Euarthropoda

**Figure 2.**
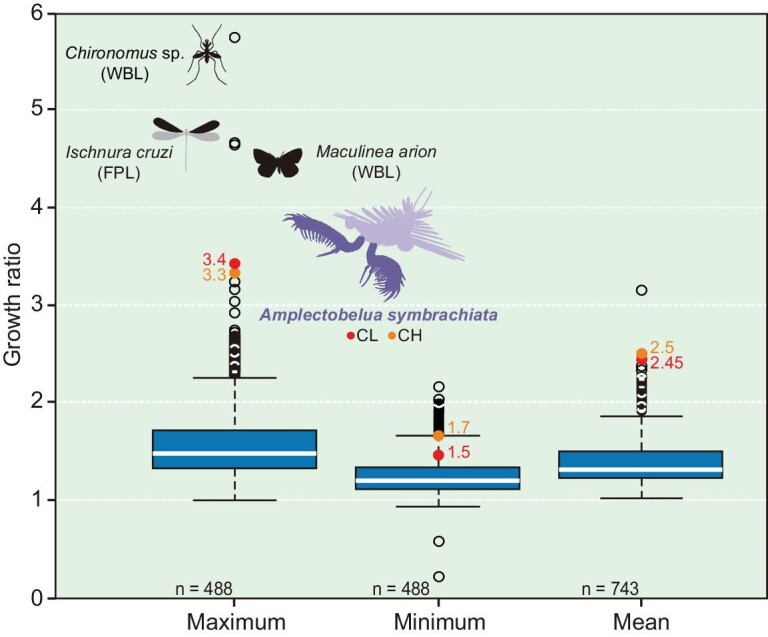
Box and whisker plot of 742 growth ratios of extant and fossil euarthropods from the literature. Box plots show comparisons of maximum, minimum and mean growth ratios between stages in total group euarthropods (data gathered from the literature). Note how *A. symbrachiata* has one of the highest maximum growth ratios of any euarthropod in the literature (except for chironomid *Chironomus*, coenagrionid *Ischnura cruzi* and lycaenid *Maculinea arion*). The diagram is collated from various body parts of a large number of modern and extinct euarthropods (see further details in Supplementary Note 4; source data provided in Supplementary Datasets 4 and 5). Abbreviations: CH, frontal appendage claw height; CL, frontal appendage claw length; FPL, forewing-pad length; n, number of specimens; WBL, whole body length.

From the literature, 742 growth ratios were gathered for extant and fossil euarthropod body parts (Supplementary Datasets 4 and 5). If the ratios between means of distributions recovered by EM are treated as growth ratios, *A. symbrachiata* has one of the highest maximum growth ratios when compared to euarthropod growth ratios identified from the literature. Only three literature values have higher proportional size increases of more than 4.0: chironomid *Chironomus* (5.74 for whole body length [32]), coenagrionid *Ischnura cruzi* (4.62 for forewing–pad length [33]) and lycaenid *Maculinea arion* (4.6 for whole body length [34]). The vast majority of growth ratios for various euarthropod body parts and the entire body length are between 1.0 and 2.0. Growth ratios exceed 2.0 in very few cases, and only seven instances (not including *A. symbrachiata*) of growth ratios above 3.0 were found documented in the literature (Fig. 2; Supplementary Datasets 4; see Supplementary Note 4 for details).

### Estimates of absolute growth parameters and mortality

**Figure 3.**
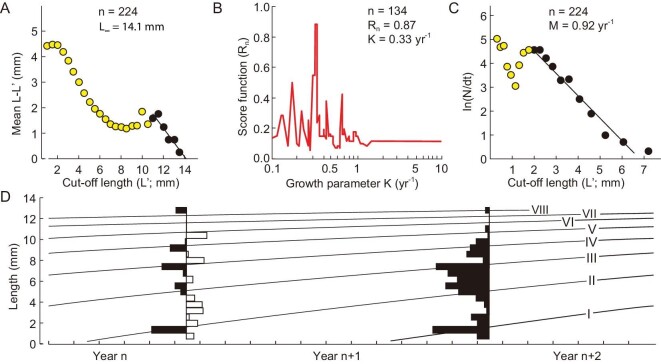
Result of the length-frequency (L/F) analyses for *A. symbrachiata.* (A) Modified Wetherall plot to estimate asymptotic length (L_∞_) using data from all sites. (B) Estimating the best K value (associated with highest R_n_ value) for L_∞_ = 14.1 mm, given the DARL L/F data from Jianshan locality (n = 134). (C) Length-converted catch curve for the estimation of instantaneous mortality (M). (D) von Bertalanffy growth curve, with L_∞_ = 14 mm and K = 0.33 yr^−1^, superposed on the original (Year n + 1) and the ‘restructured’ L/F data (Year n) (see main text). n, number of specimens; yr, year.

Length-frequency analyses were conducted on the 224 *A. symbrachiata* specimens with a complete claw. The ELEFAN approach yielded an estimated asymptotic length L_∞_ = 14.1 mm (Fig. 3A), and an estimate of K = 0.33 yr^−1^ (Fig. 3B and D). Hence the growth of *A. symbrachiata* can be represented by


(1) \begin{equation*}{{\mathrm{L}}}_{\mathrm{t}} = 14.1\left( {1 - {{\mathrm{e}}}^{ - 0.33(t - {t}_0)}} \right),\end{equation*}

where L_t_ is the length at age t (in years). The parameter t_0_, which adjusts the origin of the curve to correct for the fact that larval growth is not well described, is usually small and can be neglected here (i.e. assumed to be zero). Note also that Equation (1) can be used to describe the growth of the entire body of *A. symbrachiata*, by simply replacing the current asymptotic length by the length of the largest adult (i.e. 90 cm).

The empirical relationship t_max_ ≈ 3/K [30,35] was used to estimate a longevity of 3/0.33 ≈ 9 years. A length-converted catch curve [28,36] provided an estimate of mortality rates M = 0.92 yr^−1^, with a 95% confidence interval of 0.75–1.08 yr^−1^ (Fig. 3C). This corresponds to 60% of a population dying every year, which is rather high compared to extant marine arthropods.

## DISCUSSION

### Isometric growth in the largest Cambrian radiodont

The largest frontal appendage (Fig. 1E and Supplementary Fig. S11I) and body assemblages (Supplementary Fig. S13) of *A. symbrachiata* reported in this study allow a revised estimate (originally from ref. [37]) that the species grew up to c. 90 cm long, making it the largest Cambrian radiodont known, and the largest raptorial predator in the group (extrapolated from a claw length of 13.7 cm, and the ratio of this feature to body length from specimen JS-1917 [Supplementary Fig. S13]; see more details in Supplementary Note 8).

The isometric nature of juvenile and adult appendages means that they would have functioned in the same way, with the hypertrophied endite on the first claw podomere and robust dorsal spines strongly grasping prey items in a ‘pincer’ [18] (a redescription of this species is provided in Supplementary Note 8). Thus, as for the smallest Chengjiang radiodont, *Lyrarapax unguipsinus*, both pre-adult and adult *A. symbrachiata* individuals were raptorial predators (see Supplementary Note 9 for details), underscoring that raptorial feeding ecologies in juvenile euarthropods originated within the lower stem-group of Euarthropoda [18].

However, while the overall function of the appendage is expected to be similar, the greater size of adult *A. symbrachiata* appendages would have meant that new food sources became available. The larger size of the appendages allowed larger prey to be captured and subdued, while the greater crushing force of the larger appendages would have allowed the breaking of tougher skeletons.

Notably, the isometry in frontal appendage morphology differs from the allometry reported from the eyes of *Stanleycaris* [17] and the addition of lens rows through growth in the eyes of Emu Bay Shale radiodont [21]. Comparisons of frontal appendages in juveniles and adults (e.g. *Lyrarapax*; ref. [18]) suggest that isometric appendage growth may be conserved at least within Amplectobeluidae. Appendage measurements were not reported from *Stanleycaris* [17].

### Size-frequency data demonstrate rapid growth in *Amplectobelua symbrachiata*

Investigation of size/frequency data from different localities demonstrates rapid growth in *A. symbrachiata*—the overlapping distributions are not the result of transportation or an artefact of sampling across multiple sites.

Appendages from different localities overlap in observed size ranges (Supplementary Figs S3 and S4), and EM applied to the three sites with highest sample sizes also recovers similar means for the first two peaks (Supplementary Fig. S4; Supplementary Table S7). This, combined with the observation that euarthropod material within the size range of 25–40 mm (between peak 1 and peak 2 in claw length/frequency data) is well represented in Chengjiang deposits [9], supports the interpretation that the multimodal distribution is not an artefact of including material from multiple sites or selective transportation of material of particular sizes (for further details see Supplementary Note 3). While the sample will be time-averaged, traits will be very similar to population-level parameters [38] facilitating growth modelling of *A. symbrachiata.*

**Figure 4.**
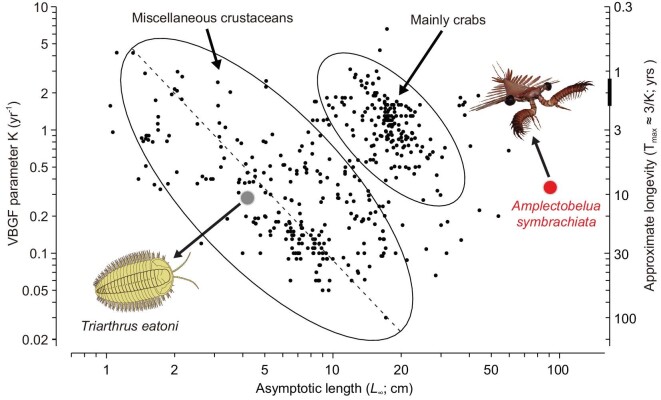
Auximetric plot with each of the small back dots documenting one of 462 pairs of L_∞_ and K values (i.e. a growth curve) in 98 species of crustaceans in SeaLifeBase (www.sealifebase.org). Note that the position of the L_∞_ and K data pair for *A. symbrachiata* is far above the large ellipsoid's main axis (dotted line), suggesting a much faster growth than recent crustaceans, as well as the Ordovician trilobite *Triarthrus eatoni* (modified from Fig. 6 in ref. [30]). VBGF, von Bertalanffy growth function.

Jointly, the growth and mortality parameters of *A. symbrachiata* estimated by ELEFAN suggest that they were extremely active animals in Cambrian ecosystems. The parameter K = 0.33 yr^−1^ initially appears to be low, however it should be borne in mind that it was estimated for an animal reaching c. 90 cm (the revised estimate of the maximum whole-body length based on our new material). Thus, at least in comparison with recent crustaceans, *A. symbrachiata* also had an exceptionally rapid growth (Fig. 4). Given that such a high growth ratio is so unusual in crown-group euarthropods (Fig. 2), it is important to consider why *A. symbrachiata* might be different. For moulting to occur, the corresponding biologically intrinsic functions are generally required to provide the prerequisites, such as the storage of nutrition used to form a new exoskeleton during the pre-moult period, recycling from the old exoskeleton, and the increase of volume and expansion of the new exoskeleton [26,39,40]. Despite growth ratios being indeed limited by physiological constraints in some species, evolution has had the opportunity to shift some physiological limits [26]. In achieving such a large growth ratio, *A. symbrachiata* may represent an extreme case in maximizing these physiological functions, without limitations introduced by a sclerotized and arthrodized body. For example, the growth ratio is, to some extent, limited by the degree to which the new cuticle is able to extend during the ecdysial expansion [26]. As a lower stem-group euarthropod, only the frontal appendages and carapace of *A. symbrachiata* were sclerotized, thus it may have been able to extend more and thus have a higher growth ratio than crown-group euarthropods, which tend to have sclerotized body parts across most of the body. During the pre-moult process *A. symbrachiata* may have been capable of storing a mass of mineral and organic matter by resorbing intensively from the old exoskeleton, food and external water column. Then these reserves would have been reabsorbed and redistributed during post-moulting to supply sufficient material basis for the newly formed exoskeleton. Furthermore, ecdysis begins with presumably intense cell division and swallowing of water to greatly expand the internal volume and surface area, and during the post-moult stage, they continue to absorb a significant amount of water, which allows for further expansion of the new exoskeleton and an increase in size [40]. Although the proportion of the whole mass of the old instar that could be reincorporated into the next instar is low (low moulting efficiency), as for the exoskeleton, a large part of the previous instar may have been consumed to recycle necessary minerals and salts to aid in the sclerotization process of the new cuticle in *A. symbrachiata*. In addition, the early fixation of the number of podomeres and isometric nature of growth would have economized the costs of production of new additional podomeres and shape alteration.

An examination of the length/frequency data suggests that this rapid growth might have been achieved over very few growth stages. If the EM-inferred normal distributions are interpreted as instars, the ratio between the mean of the first and second peaks gives a growth ratio of 3.3–3.4 for *A. symbrachiata* appendages. This is exceptionally large when compared to crown-group euarthropods (Fig. 2; Supplementary Note 4). If the growth ratio is treated as real, rather than as a result of a missing stage (Supplementary Note 5), *A. symbrachiata* would be interpreted to have developed over very few stages (as few as three stages), separated by large growth ratios early in development.

Following the Investment Principle [26], this low number of moulting events would be favoured by an animal for which moulting was inefficient or problematic. Moulting incurs the loss of resources, as not all the investment in the previous instar (including but not limited to the investment involved in the exoskeleton) can be resorbed or reincorporated (moulting efficiency). Thus, the greater the number of moulting events, the greater the number of lost resources, exacerbated by lower moulting efficiency [26]. Moulting also carries a time penalty, as it is not instantaneous, and so additional moulting events also slow down the gathering of resources [26]. Moulting also carries with it a risk of mortality, either due to problems in the moulting process itself, or from predators targeting animals during the act of moulting [22,25,26]. The subsequent post-moulting stage, during which the new expanded exocuticle begins as soft and thin, is also a perilous period for animals as they are unable to move to escape threats and would become extremely vulnerable to physical injuries, desiccation, parasitism and pathogenic infections before the new exoskeleton is sufficiently hardened [22,41,42]. Under this interpretation, a second possible benefit of employing additional stages and smaller growth ratios—increased resource accumulation—is not as important for *A. symbrachiata* as reducing the number of moulting events and/or reducing overall time in smaller growth stages.

Simulation of size/frequency data expected from instars with a growth ratio of 1.8 (square root of 3.4, the difference between the first two peaks in these data) suggests that it is plausible that a growth stage between these first two peaks existed but has not been preserved (Supplementary Note 5). ELEFAN demonstrates that *A. symbrachiata* did not display seasonal broods (Supplementary Note 7), and so this requires *A. symbrachiata* to have moulted multiple times in a year and/or for a single cohort to have had exceptionally high mortality early in life. As a gap in size/frequency data is not known in any other Chengjiang euarthropod [9,11,43], growth in *A. symbrachiata* would still be demonstrably different and more rapid than these contemporaneous euarthropods. Regardless of the interpretation of these peaks (large growth ratio or multiple moults per year), the EM and ELEFAN—although based on different premises—demonstrate rapid growth for *A. symbrachiata* (Figs 2 and 4). Similarly, the mortality estimate (M = 0.92 yr^−1^) is rather high, giving an M/K ratio of 2.8, which is near the upper limit of credible estimates in recent water-breathing ectotherms such as fish [44,45] and invertebrates (see Supplementary Fig. S8). All these data support an interpretation of *A. symbrachiata* as an extremely active Cambrian predator with rapid growth.

### Dynamics and ecological significance of rapid growth in *Amplectobelua symbrachiata*

The growth strategy of *A. symbrachiata* can be considered in light of the escalatory ‘arms race’ [46] that has been cited as one of the ecological shapers of the ‘Cambrian explosion’ [47,48]. Cambrian marine ecosystems are considered to have had high levels of predation [47,49]. High predation rates would have applied a selective pressure on these animals to grow rapidly to a large size, and becoming one of the largest animals in the ecosystem would have also provided benefits in terms of capturing prey.

From a predation-avoidance perspective, rapid growth would mean less time as a smaller animal that is more vulnerable to predation. The smallest stage of *A. symbrachiata* (mean body size estimated at 10 cm) would still have made it one of the larger animals in the Chengjiang biota. However, at this size, young *A. symbrachiata* may have still been prey for larger radiodonts, including members of the same species, as well as other large contemporaneous euarthropods.

In addition, the relative metabolic needs and energetic costs of movements (e.g. swimming, walking and flying) generally decrease with size for all animals [50]. For *A. symbrachiata*, therefore, the fast growth of the whole body (including the lateral swimming flaps) accompanied by the rapid growth of appendages would have enabled faster movement for prey capture at a reduced metabolic cost, alongside access to a wider range of prey in terms of size, and increased appendage power to subdue them.

Therefore, the unique position of *A. symbrachiata* as a large, active apex predator without a fully arthrodized or sclerotized body, in ecosystems with large amounts of predation, facilitated an active, rapid-growth strategy, potentially over very few growth stages at a growth ratio rarely seen in extant crown-group euarthropods.

## METHODS

All of the studied material is deposited at the Shaanxi Key Laboratory of Early Life and Environments (LELE) and Department of Geology, Northwest University (NWU), Xi’an, China. Fossils were photographed with a Canon EOS 5D Mark II digital camera. These morphometric data were measured from specimen digital photographs using ImageJ2. Basic statistics for variates measured are given in Supplementary Datasets 1–3. Regressions and R-squared values were calculated using PAST3. An EM algorithm was used to determine the number of normal distributions, which was achieved by normalmixEM function (mixtools package, R). Estimation of the parameters of the von Bertalanffy growth function was performed via the ELEFAN approach, as implemented using the software FiSAT II ver. 1.2.2. Full details are provided in Supplementary Notes 1, 2 and 6.

## Supplementary Material

nwad284_Supplemental_Files
