# Supplementary material for: Rapid growth in a large Cambrian apex predator
Source: Natl Sci Rev. 2023 Nov 3;11(3):nwad284. doi: 10.1093/nsr/nwad284 (PMC10833464; doi:10.1093/nsr/nwad284)
Supplement: nwad284_Supplemental_Files [file nwad284_supplemental_files.zip › Supplementary text_for_NSR_MS-2023-473_R3_2023-11-01.pdf]

Supplementary Materials for

Rapid growth in a large Cambrian apex predator

Yu Wu*, Stephen Pates, Daniel Pauly, Xingliang Zhang, Dongjing Fu*

*Corresponding authors. Email: 20219037@nwu.edu.cn (Y.W.) or djfu@nwu.edu.cn (D.-J.F.)

This PDF file includes:

- Supplementary Text (Note 1 to 9)

  Note S1. Methods
  Note S2. Recognition of stages and growth ratios
  Note S3. Specimen collection and comparison of data across deposits with largest number of samples
  Note S4. Calculation of growth ratios, comparison to other euarthropods
  Note S5. The recognition of valid stages and growth ratios in *Amplectobelua symbrachiata*
  Note S6. Using ELEFAN for estimating growth parameters and related statistics in recent and fossil water-breathing ectotherms
  Note S7. Individual peaks in trimodal distribution do not represent seasonal broods
  Note S8. Systematic Paleontology
  Note S9. Reassessment of evidence for *Amplectobelua symbrachiata* as an apex raptorial predator

- Supplementary Figs. S1 to S16
- Supplementary Tables S1 to S9
- Supplementary References
- Legends for Supplementary Datasets 1 to 8

Other supporting materials for this manuscript include the following:

- Supplementary Datasets 1 to 8 (please see the separated .xlsx, .csv and .R files)

Supporting Information Text

Note S1. Methods

Material and geological setting. The specimens documented herein were collected from 11 localities (Chengjiang, Dahaiyan, Ercai, Erjie, Haoyicun, Jianshan, Mafang, Sanjiezi, Shankou, Tanglipo, and Yunlongsi) of the Yu'anshan Member of the Chiungchussu Formation in eastern Yunnan Province, China (see more details in fig. 2 in Ref. [1]), which falls within the *Eoredlichia*–*Wutingaspis* trilobite biozone [2], Chinese local Nangaoan Stage (equivalent to Cambrian Series 2, Stage 3) [3]. In total 432 appendage specimens were used for describing the post-embryonic development of *Amplectobelua symbrachiata*. These specimens comprise 224 with complete distal articulated region, and 208 with one or more podomeres missing. The 224 complete specimens were used for investigation of isometric growth of the whole appendage and individual podomeres, and in length/frequency analyses to estimate growth and mortality parameters (Expectation-Maximization and ELEFAN analyses). The remaining 208 specimens were used for measuring heights and widths of individual podomeres, as allowed by the preservation (Supplementary Fig. S1). This means that the number of specimens measured for different appendage traits varies (see N number in Supplementary Fig. S1). All of 432 studied specimens are deposited in the Shaanxi Key Laboratory of Early Life and Environments (LELE) and Department of Geology, Northwest University (NWU), Xi'an, China.

Specimen preparation, photography, and figure preparation. All specimens analyzed in this study were gathered from split slabs of mudstones, and a few specimens were further prepared with fine needles under high magnification using stereomicroscopes. Fossils were photographed with a Canon EOS 5D Mark II digital camera, and was controlled using the EOS Utility 3.2 program for remote shooting. Images were processed in Adobe Photoshop (ver. 21.1.0) to make minor adjustments to contrast, exposure, colour balance and sharpness. Camera lucida drawings were made using a Zeiss Discovery. V12 microscope and prepared with Corel Draw (ver. 23.0.0.363). Additional figures were constructed using Inkscape 1.0.

Organization and terminology for radiodont frontal appendages. Radiodont frontal appendages consist of a series of podomeres separated by articulating membrane. The height

of the podomere refers to the distance between the dorsal and ventral margin, while length refers to the distance between boundaries separating preceding and following podomeres. Appendages can be separated into two major regions, a proximal region (here 'base' as following Refs [4–6], also termed 'peduncle' or 'shaft' in other recent works, see Refs [7–10]) where articulations between podomeres are weakly expressed, and a claw region (*sensu* Wu et al. [4,5], and Paterson et al. [6]; also called a 'distal articulated region' *sensu* Cong et al. [8], and Guo et al. [10]) which displays clear articulations between podomeres, and bears the majority of the endites. The number and arrangement of base and claw podomeres, and endites can be used to identify radiodont appendages to the family level [11]. Cp1–N refer to podomeres 1 to N of the 'claw', with numbering reflecting the proximal-distal axis (*sensu* Wu et al. [4]). In amplectobeluids like *Amplectoeblua symbrachiata*, the base region is always separated from the claw by a change in slope on the dorsal margin, and the first claw podomere (Cp1) bears a hypertrophied endite [8,12–14]. The term 'central element' and 'lateral element' refer to the central dorsal head sclerite and lateral head sclerite [15], which are equivalent to 'H-element' and 'P-element' respectively [9].

Appendage traits and measurements. We obtained six appendage traits from *A. symbrachiata* (**Fig. 1B**; for more details see Supplementary Fig. S15), using a digital caliper (0.001mm precision): (i) claw length (CL) measured from the midpoint of the proximal margin of Cp1 to the midpoint of distal margin of Cp12; (ii) claw height (CH) measured from the two endpoints of the boundary line between Cp5 and Cp6; (iii) base length (BL) measured from the midpoint of distal margin of Bp1 to the midpoint of proximal margin of peduncle; (iv) length of endite of first claw podomere (En1L); (v) length of each claw podomere(L1 to LX), and (vi) height of each claw podomere (H1 to HX). These morphometric data were measured from specimen digital photographs using ImageJ2 [16]. In a few cases small sections of the appendage were absent or covered by sediment, and the full length or height was extrapolated using ratios obtained using more complete material. Basic statistics for variates measured is given in Supplementary Datasets 1 to 3.

Statistical analyses. Changes in relative size can be described by the allometric equation $y = a*x^b$ [17], which can be expressed as a linear relationship, $\ln(y) = b*\ln(x) + \ln(a)$. We considered

measurements of claw length and each individual claw podomere length (CL and LX) as independent variables (x) and measurements of claw height, claw podomere height, and the length of the first claw endite (CH, HX and En1L) as dependent variables (y). The method of Reduced Major Axis (RMA) regression was used to estimate the relationship between the dependent and independent variables, as it handles errors in both dependent and independent variables. Natural logs of both variables were used. Regressions and R-squared values were calculated using PAST3 [18], which was also used to test the level of significance of the regressions using a permutation test. A value of b=1 (or close) indicates that variables x and y grew proportionally (i.e., isometric growth).

The claw length and heightwere plotted as histograms. The visual assessment suggested that distributions of length and height were composed of a number of overlapping normal distributions. An Expectation-maximization (EM) algorithm was used to determine the number of normal distributions, the mixing proportions, mean parameters, and standard deviations. The normalmixEM (mixtools package, R) [19,20] function was used to separate CH and CL data into 2, 3, and 4 distributions. The best model was chosen as the one with the highest (least negative) log likelihood, with all mean parameters for each group being distinct. As the same specimens were used to measure CH and CL, the number of groups had to satisfy these conditions for both CH and CL analyses (Initial values for mean parameters in mixing model in Supplementary Note 2 below).

To determine that the groupings were biological in origin, and not due to taphonomic processes transporting specimens of different sizes to different sites, CH and CL data for the sites with the highest sample sizes (Erjie, Jianshan, Mafang, Sanjiezi) were summarized, and subsequently visualised in a notched box and whisker plot (see Supplementary Note 3 below). In this type of plot, if the notches do not overlap, then there is evidence that the medians differ. The means at individual sides were compared to each other and also to the means of the recovered normal distributions following the EM algorithm.

The details of the estimation of growth parameters and related statistics are provided in Supplementary Note 6 below.

Comparative growth data in Euarthropoda. A database was compiled from the literature on the relative size of successive growth stages of total group euarthropods (Supplementary Datasets 4 and 5). Data was collected for both individual body parts, and whole specimens. Where possible, this data was separated out into individual stages, however for some data sources only an average across a number of stages was available.

The growth ratios between successive growth stages were calculated using the equation $g_i = \mu_i/\mu_{(i-1)}$, where g_i is growth ratio and μ and $\mu_{(i-1)}$ are the mean size of measurements for the ith and (i−1)th growth stages, respectively [21]. Hence, a value of 1.00 means that the size did not increase at all, while a value of 2.00 means that the feature doubled in size. The mean value (where multiple growth stages were available, or only a mean value was presented in the literature), and minimum and maximum values (where multiple growth stages were available) were determined. Boxplots of all three (mean, minimum, and maximum) were plotted, alongside *Amplectobelua symbrachiata* data presented herein.

Note S2. Recognition of stages and growth ratios by utilizing Expectation-maximization algorithm

According to the fundamental assumption from Sokal and Rohlf [22], morphological measurements from individuals from the same ontogenetic stage are often distributed normally (usually expressed as modes in histograms, or as clumps of points in bivariate plots). In many cases, clusters overlap and instar interpretations are much more subjective, or individual peaks are not clear [23]. Hunt and Chapman [24] applied maximum likelihood analysis using mixture models, to statistically identify overlapping normal distributions representing instars in fossil arthropod populations. This approach has been successfully applied in several studies [24–26]. In this study, we apply the same method to recognize overlapping normal distributions in length/frequency data of frontal appendages belonging to the radiodont *A. symbrachiata*.

Visual inspection of the histograms of claw height (CH) and claw length (CL) for all *Amplectobelua symbrachiata* specimens in this study indicated that the size/frequency data were composed of a number of overlapping normal distributions. An Expectation-maximization (EM) algorithm was used to determine the number of overlapping normal

distributions, and their mean values.

The normalmixEM function (mixtools package in R) was used to apply the EM algorithm [19,20]. Initial values for the mean parameters were estimated from the histograms of CH and CL data. As the EM algorithms can get stuck at local maxima, a number of different starting conditions were trialled, but these did not affect the resultant final mixing proportions, mean parameters, and standard deviations recovered. Example starting values for the mean parameters are given below (Supplementary Table S4).

The best model was chosen as the one with the highest (least negative) log likelihood, with all mean parameters for each group being distinct. As the same specimens were used to measure CH and CL, the number of groups had to satisfy these conditions for both sets of analyses (Supplementary Table S5).

For CH, the best model fit was for three groups (log likelihood = -151.3261). Two groups had a more negative log likelihood (-169.7733), while when CH data was separated into four groups, two of these groups had the same mean, and so the algorithm did not find four distinct groups.

For CL, the highest log likelihood was for more than three groups. Log likelihood for three groups (-514.9781) was closer to zero than forthe two-group analysis (-527.49). However, as the CH data could not be split into four groups, the three group results were chosen. Notably for distributions recovered by EM for greater than three groups in CL, a c. 3:1 ratio between the means of the first two groups was still recovered.

Recovered parameters from the EM algorithm are provided in Supplementary Table S5, and the script is provided as R file in Supplementary Dataset 6.

Note S3. Specimen collection and comparison of data across deposits with largest number of samples

All radiodont frontal appendages collected from all deposits were measured and subjected to further analysis. These deposits are within a 40 km radius and belong to the same biozone (*Eoredlichia–Wutingaspis*), and so variation due to geographic or temporal differences is likely

limited. Of 224 specimens for which both CH and CL could be measured, 1 originated from Dahaiyan locality, 2 from Ercaicun, 14 from Erjie, 134 from Jianshan, 19 from Mafang, 2 from Malong, 42 from Sanjiezi, 4 from Tanglipo and 5 from Yunlongsi (Supplementary Datasets 2).

The *Eoredlichia-Wutingaspis* biozone spans three million years, giving an upper bound for the interval of our fossil samples. However, given that exceptional fossils are generally limited to individual levels within the section, the true sampled time for our samples is almost certainly much less than three million years. According to the study of Hunt [27], time-averaging usually has limit or little effect on sample (hundreds of thousands to millions of years) variance. Therefore, the variance in our time-averaging fossil samples likely generally reflects population-level patterns. Notably, any trends (e.g., size increase through time) would serve to increase noise and overlap between instar peaks, rather than artificially create them.

Geographic or temporal differences in morphology are likely limited, and would be expected to slightly increase the variance [27] rather than introduce additional peaks in the data. However, transportation of different mean sizes of appendage to different localities is one possible way of introducing non-biological overlapping normal distributions into the length/frequency data. We investigated the impact of grouping all the samples together for EM and ELEFAN analyses by performing analyses on data from individual localities with the highest sample sizes.

To determine that the multimodal distribution of CH and CL for *A. symbrachiata* appendages were biological in origin, and not due to taphonomic processes transporting specimens of different sizes to different sites, data from the sites with the highest sample sizes (Erjie, Jianshan, Mafang, Sanjiezi) were compared.Data were visualised as scatter plots and frequency distribution histograms, in which different sites are represented by different colors. Visual inspection of these plots shows that specimens with similar size distributions are actually shared across multiple sites (Supplementary Fig. S3A). These data were also summarized (Supplementary Table S6) and subsequently visualised in a notched box and whisker plot (Supplementary Fig. S4A, B). A notched box and whisker plot visualises the median, interquartile range, maximum and minimum values for a set of data. The notches are

calculated as the median +/- 1.57, multiplied by the interquartile range, divided by the square root of the number of samples, giving an approximate 95% confidence interval for samples with more than 30 specimens (Jianshan and Sanjiezi). If the notches do not overlap, then there is evidence that the medians differ. The script for data summarization and visualisation is provided as R file in Supplementary Dataset 7. To further interrogate these data, the EM algorithm was applied to the CL data from the three sites with the largest sample sizes.

There was strong agreement in the medians and means of the four sites with the largest number of samples (Erjie, Jianshan, Mafang, Sanjiezi). The notches of all four sites overlapped, though only two (Jianshan and Sanjiezi) have high enough sample sizes for the notches to be good estimates of the 95% confidence interval. The median and mean values of all four sites were different to the recovered mean parameters from the EM algorithm (Supplementary Table S6). The EM algorithm recovered similar means for the first two groups across the three sites with the largest sample sizes (Supplementary Table S7; Supplementary Fig. S4C–E**)**. The mean of the largest group in the Sanjiezi dataset was larger than the others, and the lumped data. This is likely due to a combination of the relatively low number of larger specimens across the whole dataset and the presence of a large specimen isolated from smaller ones in the Sanjiezi data. Treating all the data together facilitates increasing the sample size across *A. symbrachiata*.

If peaks in the length/frequency histograms were due to transportation of specimens of a particular size to different sites, means of multiple overlapping normal distributions would not be expected, nor would the means and medians be expected to be comparable. It would be possible to generate a multimodal distribution for data collected from a number of sites (Supplementary Fig. S3B**)**, however, when plotted by locality the means would be expected to differ. The collected data are very different to what would be expected from this transportation.

As three overlapping normal distributions were recovered, interpreting these as the result of transportation rather than biological is challenging, due to the multimodal nature of the distribution shared across multiple sites. Lastly it is important to note that gaps in length/frequency data have not been reported from any other euarthropod from the

Chengjiang deposits (e.g., Refs [28,29]) and the size of many Chengjiang specimens (including *Isoxys auritus* carapaces; see Ref. [28]) falls within the gap between peaks 1 and 2 recovered in the *A. symbrachiata* data presented here. Thus, a model for transportation would need to be invoked that would preclude transport of *A. symbrachiata* appendages but allow transport of similar sized *Isoxys* carapaces and other euarthropod fragments.

Note S4. Calculation of growth ratios, comparison to other euarthropods

If the normal distributions are interpreted as adjacent instars, it is possible to compare the means to calculate a growth ratio between these stages. The relative magnitudes of successive growth stages were determined by dividing the mean parameter of the larger group by the mean parameter of the group immediately smaller (Supplementary Table S8). To determine how the growth ratios of CH and CL in *A. symbrachiata* compare to other total-group euarthropods, a database was compiled from the literature of euarthropod growth ratios (Supplementary Datasets 4 and 5).

742 growth ratios were found for euarthropods in the literature. The majority of growth ratios fall between 1.0 and 2.0, and only six instances were found with a maximum growth ratio above 3.0 (not including *A. symbrachiata*; Refs [30–35]). This comparative data suggests that the growth ratio for the frontal appendage of *A. symbrachiata* from stage 1 to stage 2 is extremely high for euarthropods, while the growth ratio from stage 2 to stage 3 is more comparable to other members of the phylum. The script for data comparative analysis is provided as R file in Supplementary Dataset 8. Data relating to *Spinotarsus colliseus* was excluded, following a comment by a reviewer.

Note S5. The recognition of valid stages and growth ratios in *Amplectobelua symbrachiata*

Recognizing distinct growth stages in fossil ecdysozoans can be challenging, as fluctuations in mean size across a time-averaged community will obscure size peaks relating to individual stages, and, in cases where size variation within each stage is greater than average size increase between stages, peaks will merge out of recognition [23]. Further complication may arise from males and females undertaking a different number of moults [36].

If these normal distributions are interpreted as adjacent growth stages, *A.*

symbrachiata displays very few growth stages (3) and very larger growth ratios when compared to other euarthropods (**Fig. 2** and Supplementary Note 4). Under this interpretation, the recognition of stages in *A. symbrachiata* is facilitated by the small number of growth stages, the large growth ratios between them (Supplementary Fig. S2), and the similarity in sizes of specimens across the deposits sampled from within a 40 km radius (see ref. [1]; see also Supplementary Notes 1, 2 and 3 for details).

The presence of an intervening stage living elsewhere also lacks supporting evidence, as a range of deposits were sampled and no intervening sizes were recovered, nor were any reported from a possible nursery Chengjiang deposit in Haiyan [37]. The Haiyan locality preserves fossils less sclerotized than radiodont frontal appendages, and thus likely with lower preservation potentials, measuring c. 1 mm long [38].

It is possible, however, that a growth stage was present between these two peaks but was not preserved as *A. symbrachiata* may have moulted multiple times in one year and/or there may have been mass mortality of a single cohort. To interrogate this possibility, a function *'InstarConstructor'* was created in R. This function creates a normal distribution of N specimens representing the length of specimens within instar n. The mean size depends on the growth rate between instars (G), and the mean size at instar 1. The number of specimens depends on the survival rate between instars (Sv) and how many were recruited (N).

$$I_n = G^{n-1}\ (norm(N_n\ *\ Sv^{n-1}\ ,\ \mu(I_1),\ \sigma(I_1))\ [\textit{InstarConstructor}]$$

Instars were created with G=1.8 (square root of 3.4, the ratio of the means of normal distributions 1 and 2 in the empirical data) and different recruitment rates, which simulated either poor recruitment in one instar/mass mortality of one cohort or low numbers of an instar as they had already moulted to the next stage. These instars were then combined into one length/frequency vector, subjected to EM (with the same approach as for the empirical data) to see if three peaks could be recovered from four initial instars (constructed using *InstarConstructor*). By varying the number recruited to each instar, it was possible to recover three peaks from four initial distributions (e.g., Supplementary Fig. S5**),** and thus the presence of a 'missing' instar in our data cannot be ruled out. As noted above, the lack of a gap in length/frequency data from other euarthropods in the Chengjiang means that this would still

represent an unusual feature of the growth of *A. symbrachiata*. In light of the ELEFAN analysis below, the most likely explanation would be moulting multiple times per year early in development.

Recovered parameters from the EM algorithm are provided in Supplementary Table S9, and the script is provided as R file in Supplementary Dataset 6.

Note S6. Using ELEFAN for estimating growth parameters and related statistics in recent and fossil water-breathing ectotherms

The growth of most Recent water-breathing ectotherms such as fishes, cephalopods, crustaceans, etc. can be described by the von Bertalanffy Growth Function (VBGF) as documented in multiple publications; see FishBase (www.fishbase) for fishes and SeaLifeBase (www.sealifebase.org) for various invertebrates. Also, Pauly et al. [39] demonstrated that the VBGF, although it expresses growth as a continuous function, can be used to describe the stepwise somatic growth of water-breathing ectotherms (WBE) resulting from moulting, as occurs in crustaceans, a major group of arthropods. The VBGF has also been applied successfully to the length-frequency data from an Ordovician trilobite, *Triarthus eatoni* [40], and thus the interest in its use for describing the growth of the radiodont *Amplectobelua symbrachiata*.

The standard VBGF has the form:

$$L_t = L_\infty \cdot (1 - e^{-K \cdot (t - t_0)}) \quad \text{`} \qquad \ldots 1)$$

where L_t is the mean length at age t of the WBE in question, L_∞ their (mean) asymptotic length, i.e., the mean body length (or any other linear measure of size) they would be reached after a very (i.e., an infinitely) long time, K a coefficient of dimension time^{-1} (here: yr^{-1}), expressing how rapidly the asymptotic size is approached (i.e., K is not a growth rate), and t_0 corrects for the fact that the VBGF does not capture well the growth of larval and early post-larval WBE [41]. The parameter t_0 cannot be estimated from length-frequency data alone, but fortunately, it is often not required or usually small enough to be neglected. Several other versions of the VBGF exist, including for seasonal growth, and growth in weight, but they are not likely to be useful for describing the growth of fossils (see Supplementary Materials in Ref. [40], available

at Zenodo Digital Repository: https://doi.org/10.5281/zenodo.6640357).

The growth parameters L_∞ and K can be estimated sequentially. First, L_∞ can be estimated by a plot proposed by Wetherall et al. [42]. This method for estimation of L_∞ from length-frequency data representative of a population of WBE does not require 'growth' data, or estimates of K to be available. Rather, it consists, when length-frequency data (L/F) are available, of plotting successive mean lengths (L_i) against the lengths (the lower limit of the 'bins' of the L/F data) from which the means are computed (or 'cutoff length; 'L_i'), i.e.,

$$Li = a + b \cdot L_i' \qquad \ldots 2)$$

where, for the straight segment of the plot (i.e., including only fully recruited and selected length classes), $L_\infty = a/(1\text{-}b)$. However, we used here, for easier visualization (see Supplementary Fig. S6) a modified version of Equation 2 was used, i.e., a 'Modified Wetherall Plot' [43], where L_i - L_i' is plotted against L_i, and L_∞ = - a/b, and M/K = - (1+b)/b, with M being the instantaneous mortality rate, and the M/K ratio usually have a relatively narrow range in Recent WBE (see below).

Once L_∞ is estimated, K can be estimated via a 'scanning for K' routine (as built in in the ELEFAN software package known as FiSAT; see Ref. [43]), which identifies the estimate of K which maximizes the fit (expressed as 'R_n') of the VBGF to a set of 'restructured' L/F data arranged in a suitable number of 'bin,' i.e., length classes a given width. Here, the width of the length classes matters, and FiSAT has a routine to change their width, which allow the identification of the class width allowing for the best fit of growth curves to the restructured L/F data.

'Restructuring' L/F data in the context of ELEFAN consist of applying a running average (over 5 length classes) to these data, which mimics a high-pass filter with the original histogram being either above the running average line ('peaks') or below that line ('troughs'). The original histogram values are then divided by the corresponding running average values and 1 is subtracted (and some other minor adjustments are performed). The results are positive 'peaks' (black) and negative 'through' (white), with the former generally representing

age groups identified without assumption of normality, or spawning/recruitment periodicity (Supplementary Fig. S6).

Fitting the VBGF to a set of restructured L/F data when L_∞ is known then consists of superposing thousands of growth curves with different K and different starting points (which replace the parameter t_0), and retaining the curve which 'hits' most peak while avoiding throughs. This is expressed in the fraction $R_n = 10(^{ESP/ASP})/10$, where ESP (the 'Explained Sum of Peaks') represents the peaks 'hit' by the VBGF, and ASP (the 'Available Sum of Peaks') represent all the peaks available in a set of restructured L/F data (see Supplementary Fig. S7), with the highest R_n score identifying the best estimates of the VBGF's parameters (Ref. [41], and see Supplementary Fig. S7). The software used here to implement the ELEFAN method was FiSAT, documented in Gayanilo et al. [44] (but see also Refs [45,46]).

As shown in Supplementary Fig. S7, restructured L/F samples is used are plotted and 'used' repeatedly at annual intervals, under the assumption that the spawning/recruitment events and the growth pattern remain self-similar from year to year. This assumption can be relaxed when abundant L/F data covering many successive years are available [47], which are not likely to ever occur with L/F data on fossils. The same applies to seasonally oscillating growth, which does occur in Recent arthropod [39] and likely occurred in long-extinct one as well [40], but which requires L/F data set from different seasons (or better: months) within years, which are not likely to be available for ancient fossils.

Instantaneous mortality (M) is defined for any time period, e.g., a year, as $M = -\ln(N_{i+1}/N_i)$, where N_i and N_{i+1} the number of WBE in a cohort, i.e., animals born or hatched in the same year, and experiencing the same environmental conditions, etc. Under the assumption of the underlying population being in equilibrium, i.e., not growing of declining during the sampled period (an unavoidable assumption for fossil L/F), M can be estimated from the age distribution in the population, which itself can be inferred from L/F data. This leads to length-converted catch curves, where M can be inferred from the (descending) slope (b; with sign changed) of the plot

$$\ln(N/\Delta t) = a - b(t')$$

where N is the number of WBE in each length class, Δt is the time needed for the WBE in question to growth from the lower limit of that class to its upper limit, and t' is the relative age ('relative' because t_0 is not required in catch curves) corresponding to the mean of the upper and lower limits of each class [41].

One key issue with length-converted catch curves (which they share with age-based catch curve; see Ref. [48]) is the bias due to incomplete recruitment and selection and of younger individuals, and de-recruitment of larger, older individuals. The former processes lead to the abundant, younger length and age groups not being (fully) represented in a catch curve because they may not have been 'recruited' to the sample population (e.g., because they lived in another habitat), or they are under-sampled because they are too small, while the latter process is often due to large/older adults moving to deeper water in order to reduce their metabolic rate, which becomes limiting to their growth and activity levels [39,49,50].

Thus, the most informative section of a catch curve (length-converted or not) is its 'central' part, based on abundant, fully recruited and selected part of a population. Finally, as this section is identified by the straightness of the alignment of the points representing age (or length classes), the computed standard error of the slope of the regression fitted to these points will underestimate the real uncertainty of the estimate of mortality (i.e., the absolute value of the slope).

The modified Wetherall Plot described above has similar issues, and while it is suitable for estimating L_∞, it often predicts M/K ratio which are unrealistically high, i.e., well above the values of 2.0–2.5 that are maximally encountered in fishes [51–53 and other WBE (see Supplementary Fig. S8).

The above remarks, and the detailed discussion of different growth curves in the Supplementary Materials of Pauly and Holmes [40] should be sufficient to justify the application of the ELEFAN methodology to contrast-rich length-frequency data of fossil WBE.

Note S7. Individual peaks in trimodal distribution do not represent seasonal broods

The ELEFAN approach and software were developed to estimate, from length-frequency (L/F)

data, the parameters of the von Bertalanffy Growth Function (VBGF, widely used by fisheries scientists and marine biologists) of tropical fish, which often lack the seasonal marking (or annuli) allowing the age of temperate fish to be inferred, or which are lacking in arthropods (e.g., shrimps) from all latitudes.

While temperate, longer-lived fish of higher latitude generally have one well-defined spawning season per year (often in spring), shorter-lived tropical fish and invertebrates usually have two broad spawning peaks separated by 5 (or 7) months, generally in the post-monsoon seasons [49]. However, when examining raw L/F data from tropical fish or invertebrates, it is usually difficult to assess if their peaks or 'broods' were produced by a population with one or two spawning events per year. The ELEFAN approach and software were designed such as to completely circumvent this and related issues. Thus, the 'restructuration' of the available L/F data enables ELEFAN to identify 'peaks' (usually representing age groups) without any assumption as to the ages they correspond to, and the spawning frequency in the studied population.

The strong assumptions that ELEFAN relies are rather 1) that the growth of the fish or invertebrates in question is asymptotic, i.e., rapid at first, then declining with length (such as expressed by the VBGF), and 2) the growth curve parameters do not change from year to year. Given these two assumptions, ELEFAN traces a multitude of growth curves over the restructured L/F data repeated year after year, and records for each growth curve the number of peaks (= positive 'points') that the curve hits, while avoiding the 'troughs' between the peaks (= negative 'points') (Supplementary Fig. S9).

ELEFAN then computes, for each curve, a 'goodness of fit' index, $R_n = 10^{(ESP/ASP)}/10$, where ASP is the Available Sum of Peaks in the restructured L/F dataset and ESP is the Explained Sum of Peaks, i.e., the peaks whose position is 'explained' by being along the trajectory of a growth curve. The parameters of the curve with the highest R_n value are then retained.

In practice, this amount to only scanning a wide range of values of the K parameter of the VBGF (e.g., 0.1 to 10 yr^{-1}), because the other important parameter of the VBGF, asymptotic

length (or L_∞) can be inferred from the largest specimen in L/F data that include adults. As for the 3rd parameter of the VBGF, t_0, it is replaced within ELEFAN, by an arbitrary 'starting point' and has no impact on the estimation of K.

This procedure, which identifies the best growth curve that fits a L/F dataset is completely insensitive to the presence of 'secondary' growth curve(s), whose only effect is to reduce the R_n value of the best curve (because secondary growth curves increase the ASP). A putative secondary growth curve generates peaks that increase the ASP, but whose positions is not explained by the best growth curve (Supplementary Fig. S9). Thus, its ESP remains low, which would lead to a low R_n. In our case, the fact that R_n is very high (0.9) implies that only one spawning event (or 'brood') occurred per year.

Note S8. Systematic Paleontology

Superphylum **Panarthropoda** Nielsen, 1995 [54]

Order **Radiodonta** Collins, 1996 [55]

Family **Amplectobeluidae** Pates, Daley, Edgecombe, Cong, Lieberman, 2021[12]

Genera included. *Amplectobelua* (Hou, Bergstrom & Ahlberg, 1995 [56]) (type genus); *Lyrarapax* (Cong, Ma, Hou, Edgecombe & Strausfield, 2014 [57]); *Ramskoeldia* (Cong, Edgecombe, Daley, Guo, Pates & Hou, 2018 [14]); *Guanshancaris* (Zhang, Wu, Lin, Ma, Wu & Fu, 2013 [58]). Possibly *Laminacaris* (Guo, Pates, Cong, Daley, Edgecombe, Chen & Hou, 2019 [10]).

Emended diagnosis (adapted from Pates et al., 2021 [12]). Radiodont with frontal appendages with dorsal kink separating base and claw; slightly enlarged to hypertrophied endite on first claw podomere; tripartite carapace composed of ovoid central element and paired lateral elements of a similar size to central element; reduced 'neck' region composed of three to four small flaps; body flaps bear strengthening rays on anterior part only.

Remarks. Although the internal relationships within Radiodonta are not well resolved, numerous studies using distinct character datasets have recovered *Lyrarapax* as the sister taxon to *Amplectobelua*, and thus the former should be considered as part of family

Amplectobeluidae [9,57]. By the same reasoning, *Innovatiocaris*? sp. should also be considered an amplectobeluid, as it has also been consistently recovered with *A. symbrachiata*, such as the presence of hypertrophied first claw endite and long fifth claw endite (see fig. 13 in Ref. [62]).

Diagnosing the family Amplectobeluidae is complicated, as numerous characters are shared between some but not all these taxa, and some features common to all amplectobeluids are also shared with select other radiodonts, in particular anomalocaridids.

Within the frontal appendage, a dorsal kink between the base and claw is known from all amplectobeluids, and also other radiodonts such as *Houcaris saron* [5]. The presence of a hypertrophied endite is known from *Amplectobelua, Lyrarapax*, *Guanshancaris*, as well as *Innovatiocaris*? sp. [8,57,59,62] (see also in Supplementary Figs. S10 and S11), however the endite on the first claw podomere (Cp1) in *Ramskoeldia* and *Innovatiocaris*? *multispiniformis* is only slightly enlarged, and is instead more comparable to anomalocaridids such as *Anomalocaris canadensis* [14,62,63]. Another, more subtle feature – the presence of a longer endite on the fifth claw podomerethan the third – unites *Amplectobelua, Ramskoeldia*, *Innovatiocaris*? sp., *Innovatiocaris*? *multispiniformis* and *Guanshancaris kunmingensis*, but is lacking in *Lyrarapax* [8,14,58,62,64]. This feature is also possibly present in a radiodont of uncertain affinities known solely from frontal appendages, *Laminacaris chimera* Guo et al. 2019 [10], which has been recovered as an amplectobeluid in some phylogenetic analyses [9] but not all (e.g., Refs [9,65]). *Laminacaris* also displays a hypertrophied endite on the first claw podomere, but lacks a dorsal kink between base and claw.

A tripartite carapace with ovoid central element and small lateral elements unites *Amplectobelua, Lyrarapax* and *Ramskoeldia* [8,14,57], however the carapace is currently unknown in *Guanshancaris kunmingensis* [58,59,64]. The feeding apparatus in the head region is more variable. The presence of gnathobase-like structures and tuberculate and smooth plates is only known in *Amplectobelua, Ramskoeldia* and Radiodont C [8,14,66]. *Guanshancaris* and *Lyrarapax* instead possess oral cones with four large plates separated by smaller plates, but apparently lack gnathobase-like structures [64,67,80].

The body region is best known in *Amplectobelua symbrachiata* and *Lyrarapax,* only flaps are known in *Ramskoeldia* and no post-cephalic morphology is currently known for *Guanshancaris.* A reduced neck region is common to *Amplectobelua* and *Lyrarapax,* however it is also known from other radiodonts including anomalocaridid *Anomalocaris canadensis* and hurdiid *Peytoia nathorsti* [8,57,63,68]. Similarly, the presence of strengthening rays exclusively on the anterior part of the flap is known in all three amplectobeluids from which flap material is known, however this feature is again shared with *Anomalocaris canadensis* and *Peytoia nathorsti* [63,68].

The characters chosen in the diagnosis reflect those found in all amplectobeluids, even if some are also known in additional radiodonts (e.g., dorsal kink in frontal appendage, strengthening rays on anterior half of flaps) in combination with those known from all amplectobeluids where features are preserved (e.g., tripartite carapace of small lateral elements adjacent to ovoid central element). The presence of a hypertrophied endite on the first claw podomere is also retained in the diagnosis, as this is the most recognizable feature of amplectobeluid frontal appendages, is exclusive to the genus, and even *Ramskoeldia* appendages display a small degree of hypertrophy on this first claw endite. Other features, such as the presence of gnathobase-like structures and the fifth claw podomere bearing a longer endite than the third, are still useful in identifying amplectobeluids, but are not retained in the diagnosis as they are not present in all amplectobeluids. Instead, these characters should be considered diagnostic of lower taxonomic levels, such as genera.

Genus ***Amplectobelua*** Hou, Bergström & Ahlberg, 1995 [56], amended.

Type species. *Amplectobelua symbrachiata* Hou, Bergström & Ahlberg, 1995, from the Yu’anshan Member, Chiungchussu Formation (Cambrian Series 2, Stage 3), Yunnan Province, China [56].

Emended diagnosis (adapted from Cong et al. 2017 [8])**.** Amplectobeluid with frontal appendage bearing pairs of simple spine-like endites devoid of auxiliary spines; En1 paired and exceptionally enlarged; Cp2 to Cp11 are tall rectangular; frontal carapace composed of central element and paired lateral elements connected by rod-shaped plate; mouth apparatus

composed of smooth and tuberculate plates; three pairs of gnathobase-like structures associated with reduced transitional flaps immediately posterior to mouthparts.

Remarks. New material reveals that the exceptionally long En1 of *A. symbrachiata* is paired rather than unpaired (Supplementary Fig. S11C and E), as previously recognized for *A. stephenensis* [13]. Thus, a paired En1 is a common character in this genus. Claw podomeres, with the exception of the most proximal and distal ones, are tall and rectangular in this genus. These features are added to the diagnosis (see above).

Amplectobelua symbrachiata Hou, Bergström & Ahlberg, 1995 [56]

(**Fig. 1E–G and** Supplementary Figs. S10–S13)

1992 *Anomalocaris trispinata*; Shu *et al.*, pl. 1, fig. 2 [69].

1994 New anomalocaridid animal 2 from Chengjiang; Chen *et al*. p. 1305, figs. 3 [70].

1995 *Amplectobelua symbrachiata*; Hou *et al.*, pp. 176–177, figs. 14–15 [56].

1996 *Amplectobulua symbrachiata*; Chen *et al.*, pp. 199–200, figs. 267–272 [71].

1997 *Amplectobelus sumbrachiata*; Chen & Zhou, pp. 79–80, figs. 125–128 [72].

v. 1999 *Amplectobelua symbrachiata*; Hou *et al.*, p. 68, figs. 83–84 [73].

1999 *Amplectobelua symbrachiata*; Luo *et al.*, pl. 16, figs. 2–6 [74].

1999 *Hipopotrum spinatus* Luo, Hu in Luo *et al.*; Luo *et al.*, pl. 27, fig. 7 [74].

2002 *Amplectobelua symbrachiata*; Chen *et al.*, pl. 14, figs. 3–4 [75].

v. 2004 *Amplectobelua symbrachiata*; Hou *et al.*, p. 97, fig. 15.3 [76].

2004 *Amplectobelua symbrachiata*; Chen, pp. 302–304, figs. 483, 485–487 [77].

2005 *Anomalocaris* sp.; Zhao *et al.*, pl. 4, fig. 8 [78].

v. 2014 *Amplectobelua symbrachiata*; Cong *et al.*, extended data fig. 1b–d [57].

v. 2017 *Amplectobelua symbrachiata*; Hou *et al.*, p. 157, fig. 19.3 [3].

2017 *Amplectobelua symbrachiata*; Zeng *et al.*, p. 23, fig. 18e, f [67].

? 2017 undetermined taxon (NIGPAS 162524); Zeng *et al.*, p. 16, fig. 12 [79].

2017 *Amplectobelua symbrachiata*; Cong *et al.*, pp. 4–13, figs. 1–8, 9A, 10 [8].

2018 *Amplectobelua symbrachiata*; Liu *et al*.; supplementary fig. S3A [80].

New material examined. Total of 432 specimens from the Chiungchussu Formation, eastern Yunnan, south-west China. JS-0244AB, SJZ-0020, JS-0152AB, SJZ-139AB, SJZ-171, SJZ-204AB, SJZ-281, SJZ-305, SJZ-451.

Type material. NIGPAS 115346 (holotype), a juvenile specimen with nearly complete frontal appendages, a head shield and P-elements [70].

Occurrence. Cambrian Series 2, Stage 3 (Chinese local Nangaoan stage), Yu'anshan Member, Chiungchussu Formation, *Eoredlichia*–*Wutingaspis* trilobite biozone (Ercai, Erjie, Jianshan, Mafang, Sanjiezi, Shankou, Tanglipo, Yunlongsi, Chengjiang, Malong), eastern Yunnan, south-west China.

Emended diagnosis (adapted from Cong et al. 2017 [8]). *Amplectobelua* with sturdy frontal appendages consisting of three base podomeres (Bp1–3) and 12 claw podomeres (Cp1–12); claw podomeres articulate at dorsal pivot joints; En1 paired and hypertrophied, between one-third to half as long as the length of the distal articulated region, and carrying two lateral spines branching from its most basal part; En1 to En11 attach ventrally to podomeres by sockets; podomeres and endites decrease in size distally except for Cp5 and En5; endites alternate in size; from Cp2 to Cp11, podomeres nearly three times as tall as long ; dorsal spines present on Cp9 to Cp12; dorsal spines on Cp11, 12 significantly larger than those on Cp9, 10; Cp12 semicircular and bears two pairs of terminal spines; central carapace element oval, with posterior edge nearly straight; lateral elements ovoid, with rod-shaped plate that joins them divided by triangular central region; gnathobase-like structures with small scale-like nodes on the blade and four pairs of robust spines along the distal margin; trunk flaps display strengthening rays on anterior part only.

Description. Frontal appendages of *Amplectobelua symbrachiata* preserved in isolation are quite common in the Chengjiang biota (e.g., Supplementary Fig. S10). The largest complete specimen is 19.64 cm in length (Supplementary Fig. S11I). The appendage consists of 15 podomeres, including 3 base podomeres (Supplementary Fig. S10D and S11I) and 12 claw podomeres (Supplementary Figs. S10 and S11). The base (Ba) and claw articulate against each

other on the dorsal surface. The angle between the axis of base and claw varies from 100° to 180° (Supplementary Figs. S10A–D and K, and S11A, H and I). A large triangular hinge membrane is located on the distal-most base podomere (Bp1), and adjoins the boundary between Bp1 and proximal-most claw podomere (Cp1) (Supplementary Fig. S12A–F). The claw of frontal appendages varies in length (CL) from 0.91 cm to 13.71 cm and height (CH) from 0.13 cm to 2.56 cm (Supplementary Table S1). The height of podomeres decreases distally, and the length of podomeres decreases distally except for Cp5 (Supplementary Figs. S10D and E, S11A and B, and S14). In JS-0244, the base is elongate and sub-rectangular in lateral outline (Supplementary Figs. S10D and S11I). The appendage generally curves ventrally. All podomeres, with the exception of Cp1 and Cp12, are relatively narrow in outline and the average height:width ratio is 2.92 (Supplementary Table S2). Cp1 is enlarged and bears paired exceptionally long curved endites (En1). The claw podomeres articulate by means of dorsal pivot joints (hollow arrows in Supplementary Figs. S10F and H, and S11A). The triangular shape separating the podomeres (am in Supplementary Fig. S12B and H) resembles the flexible cuticle or arthrodial membrane described in other radiodont taxa, including some *Lenisicaris* and *Anomalocaris* species (e.g., *Lenisicaris pennsylvanica*, fig. 7.1–7.4 in Ref. [81]; *A. canadensis*, fig. 13.2, 3, 5, 7 in Ref. [63], and fig. 1 in Ref. [82]), but have not yet been described in previous work in this genus [8,13,56]. The angle subtended by this area varies within a maximum of about 20° (Supplementary Fig. S11H) and decreases in proportion to the curvature of the appendage, which is to be expected for an articulation zone. In general, the size of endites on both even and odd claw podomeres decreases distally except on Cp5 (Supplementary Fig. S10B–D). The En1 carries three relatively smaller lateral spines projecting from near the base of the En1 (Supplementary Fig. S10I). Frontal appendages usually display a series of dark round pigments on the ventral surface of each claw podomere, these structures are interpreted as articulatory sockets, facilitating attachment of endites to the podomere (Supplementary Fig. S10I–L). In the counterpart of specimen SJZ-139, each of Cp10 to Cp12 bears a dorsal spine, and the dorsal spines of Cp11 and Cp12 are much larger and curve distally over the end of the appendage (Supplementary Fig. S10C). SJZ-204 shows that the Cp11 also bears a smaller secondary dorsal spine (sds in Supplementary Fig. S10I and J). In

addition, Cp12 bears a pair of terminal spines (ts in Supplementary Fig. S11E and F) and smaller secondary terminal spines at the distal end (sts in Supplementary Figs. S10I, and S11E–G), which together with the dorsal spines of Cp11 and Cp12 form a heavily armoured distal termination. In the part of articulated assemblage specimen (JS-1917), nine paired lateral body flaps could be identified, although they only preserved the basal part with exception for the second flap (Supplementary Fig. S13A, B). Moreover, specimen JS-1917 also shows paired frontal appendages, central element and partial possible lateral element of head sclerite complex, and fragments of gnathobase-like structure, eyes and setal blades (Supplementary Fig. S13). This specimen measures c. 110 mm (sag.) from the anterior margin of the appendage to the posterior tip of the body, with the distal articulated region of appendage being c. 17 mm long.

Remarks. Based on the new specimens, we have revealed several new characters of frontal appendage of *Amplectobelua symbrachiata*, including the presence of articulatory sockets at the base of endites, secondary dorsal spine on Cp11, secondary terminal spines, as well as the hinge membrane on the distal-most base podomere. Cong et al. [8] illustrated a single specimen showing a triangular 'short podomere' preceding the proximal-most claw podomere (fig. 4c in Ref. [8]), and interpreted this special structure as a result of taphonomic artefacts or a soft arthrodial membrane. We here consider this structure is not the result of taphonomic artefacts due to it could be identified in several appendage specimens (Supplementary Fig. S12B, D and F), and interpreted it as hinge membrane located on the distal-most base podomere. Thus, the place where Cp1 and Bp1 meet constitutes a hinge joint, which may be functionally analogous to the carpal joints of cheliped of extant crabs (Supplementary Fig. S12G), allowing a greatly horizontal movement of the distal claw part of appendage relative to base with the appendage tips meeting at the midline. The size of hinge membrane varies with the degree to which the hinge joint is bent (see Supplementary Fig. S12B, D and F; fig. 4c in Ref. [8]). The hinge membrane is only visible in the appendage specimens in lateral-inner view, whereas is hardly identified in the specimens in lateral-outer view. This structure has also been potentially identified in some other radiodont taxa, such as *Houcaris saron* and *Ramskoeldia consimilis* [62]. The ratio of body length to claw length is at least c. 6.5 (Supplementary Fig.

S13, and fig. 3 in Ref. [70]), allowing a revised estimate for the largest body size reaching 90 cm.

Daley and Budd [13] noted that *Amplectobelua symbrachiata* differs from the only other congener, *A. stephenensis* from the Burgess Shale, in the number of podomeres on the frontal appendage (15 in *A. symbrachiata* versus 12 in *A. stephenensis*) and the endite morphology, especially the En1. Daley and Budd [13] observed that that the podomere that bears the largest endite in the two known species is different, i.e., podomere 4 in *A. symbrachiata* versus podomere 1 in *A. stephenensis,* and this difference might be caused by preservation (*A. stephenensis* missing first three podomeres) or reflect a biological difference. As more recent studies have noted, often only the claw podomeres are preserved in many radiodont frontal appendages. Furthermore, a specimen from the Wheeler Formation bearing numerous similarities to *A. stephenensis* possibly preserved some of the base podomeres (see fig. 2A, B in Ref. [83]). Thus, currently known *A. stephenensis* frontal appendage specimens likely only preserve the claw and did not preserve base as in *A. symbrachiata*. The podomere bearing the large endite in *A. stephenensis* is the first claw podomere, and homologous to Cp1 of *A. symbrachiata*. The number of peduncular podomeres in *A. stephenensis* remains unknown.

Note S9. Reassessment of evidence for *Amplectobelua symbrachiata* as an apex raptorial predator

Amplectobelua symbrachiata is interpreted as an apex macrophagous predator because of its large body size, spinose feeding apparatuses (including robust spinose frontal appendages, and gnathobase-like structures close to mouth) and strong capabilities for active swimming (Supplementary Figs. S15 and S16). The claw of *A. symbrachiata* frontal appendages was equipped with hypertrophied endite on Cp1 and a series of spiny endites that is typically attributed to a grasping function [13]. The newly revealed articulatory socket at the base of hypertrophied first claw endite (Supplementary Fig. S10K and L) indicates that it may have functioned as the movable part of a claw, and thus serve as a structure to greatly enhance the function of picking up and fixing prey. The well-developed arthrodial membranes between podomeres would allow a strong curvature of appendage and provide sustained pressure on

prey, indicating that they could tightly hold large animals. In addition, the presence of short spiny endites on Cp2 to Cp11, and in particular the robust dorsal and terminal spines would make them suitable for piercing armored prey items. The presence of hinge membrane (Supplementary Fig. S12; fig. 4c in Ref. [8]) would makes the distal-most base podomere (Bp1) as a structure being functionally analogous to the carpus of chelipeds in crabs, allowing an extremely wide range of horizontal adduction and abduction for the claw to easily deliver the prey to the mouth. All these traits coevolved to create a powerful force and optimise functionality of the large frontal appendages, and optimise potential metabolic or locomotor costs. Another inferred feeding structure, the gnathobase-like structures close to the mouthparts, bear striking similarities to the gnathobase that widely known as the basal part of appendages in euarthropods, indicating that it could be used for manipulating and masticating food items (possibly including shell crushing), possibly functioning in a similar way to mammalian molarsa [80,84].

In order to effectively capture quickly moving, larger and more nutritious preys, *Amplectobelua symbrachiata* would also have relied on its size, swimming ability, and sensory system composed of large compound eyes. The habitus of *A. symbrachiata* indicates it was an excellent swimmer, as suggested by its powerful swimming body flaps (allowing for metachronal swimming). Past hydrodynamic studies have shown that optimal swimming performance was found to occur when the lateral overlapping body flaps acted as a single flap, as in many batoid fishes [85,86]. The paired large, lateral stalked compound eyes would have provided acute stereoscopic vision, facilitating size discrimination, and shape resolution of possible prey [87,88]. Acute vision in predatory arthropods favors tracking, capturing, or ambushing prey [89]. This type of eyes contrasts to the sessile and non-stalked eyes in *Echidnacaris briggsi* [6,90]. Compared to the large head carapace complexes of hurdiid radiodonts [9,65,91,92], as well as cephalothoracic carapaces in some large bivalved arthropods [88], the head carapaces of *A. symbrachiata* were much smaller, suited for its active, nektonic lifestyle, since such feature would reduce weight and drag during moving [93].

Supplementary Figures 1–16

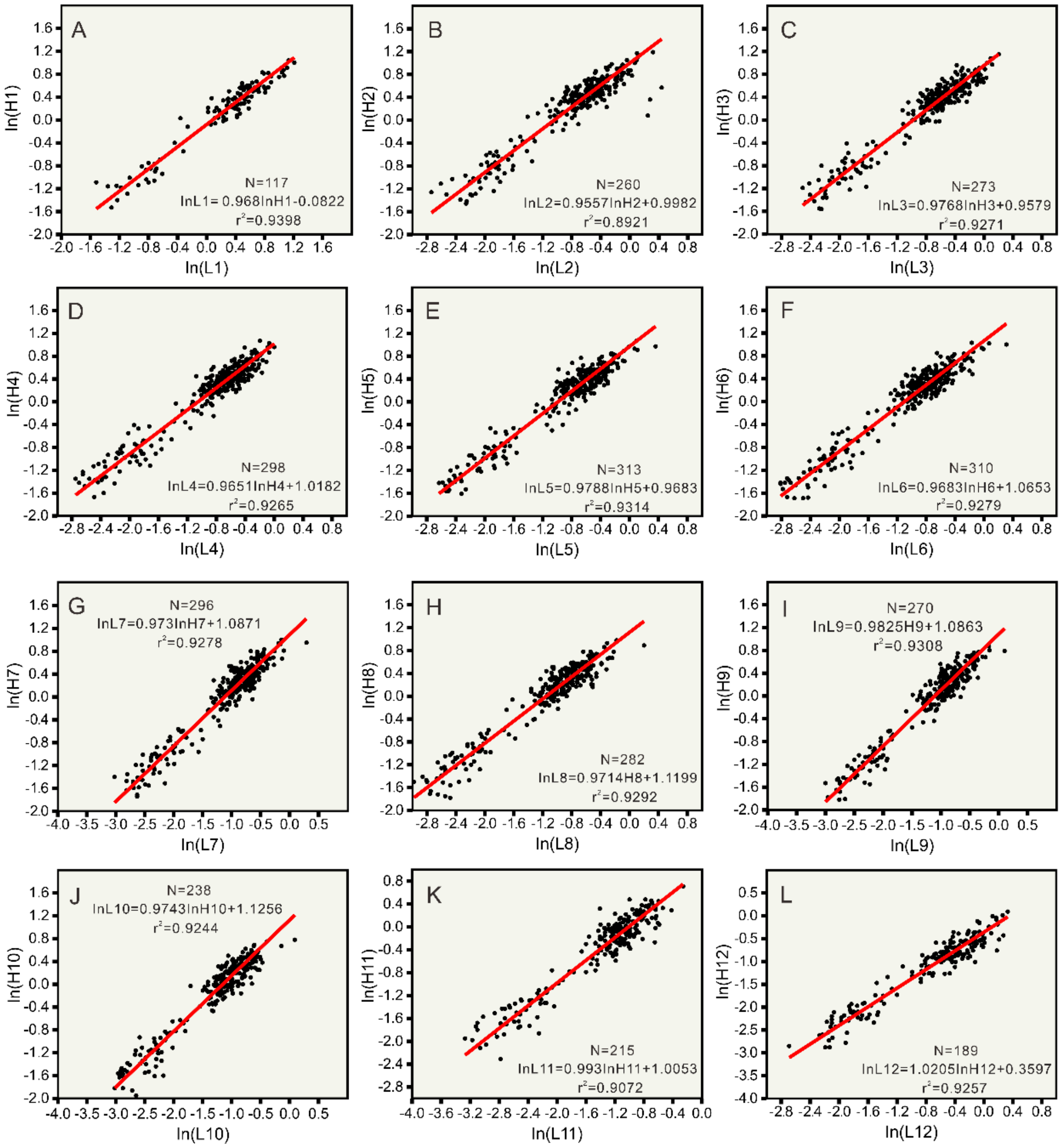

Supplementary Fig. S1. Scatter plots of the ontogenetic relationship between length and height of each podomere in the distal articulated region from Cp1 to Cp12. (A) ln length of Cp1 (lnL1) regressed against ln height of Cp1 (lnH1). (B) ln length of Cp2 (lnL2) regressed against ln height of Cp2 (lnH2). (C) ln length of Cp3 (lnL3) regressed against ln height of Cp3 (lnH3). (D) ln length of Cp4 (lnL4) regressed against ln height of Cp4 (lnH4). (E) ln length of Cp5 (lnL5) regressed against ln height of Cp5 (lnH5). (F) ln length of Cp6 (lnL6) regressed against ln height of Cp6 (lnH6). (G) ln length of Cp7 (lnL7) regressed against ln height of Cp7 (lnH7). (H) ln length of Cp8 (lnL8) regressed against ln height of Cp8 (lnH8). (I) ln length of Cp9 (lnL9) regressed against ln height of Cp9 (lnH9). (J) ln length of Cp10 (lnL10) regressed against ln height of Cp10 (lnH10). (K) ln length of Cp11 (lnL11) regressed against ln height of Cp11 (lnH11). (L) ln length of Cp12 (lnL12) regressed against ln height of Cp12 (lnH12). Measurements in cm.

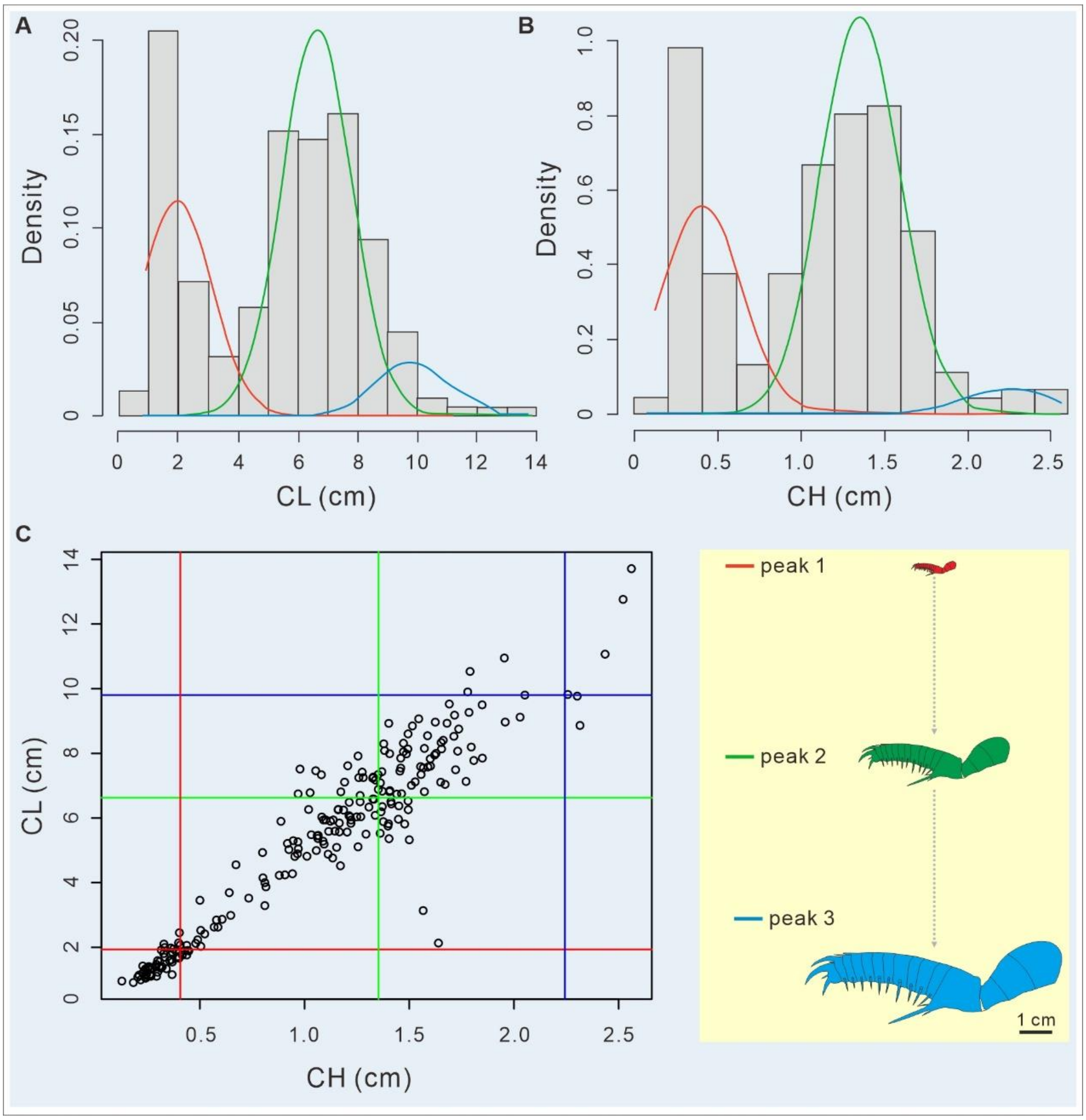

Supplementary Fig. S2. Histograms and scatter plot of claw size distributions. Histograms showing claw length (A) and claw height (B) data of *Amplectobelua symbrachiata* frontal appendages. Overlain results from Expectation-maximization (EM) algorithm, showing three overlapping normal distributions – red relates to stage 1, green stage 2, blue stage 3. Source data provided in Supplementary Datasets 1–3. Means of normal distributions recovered overlain on scatter plot of claw height vs. claw length (C) with relative mean sizes of each appendage displayed, to same scale.

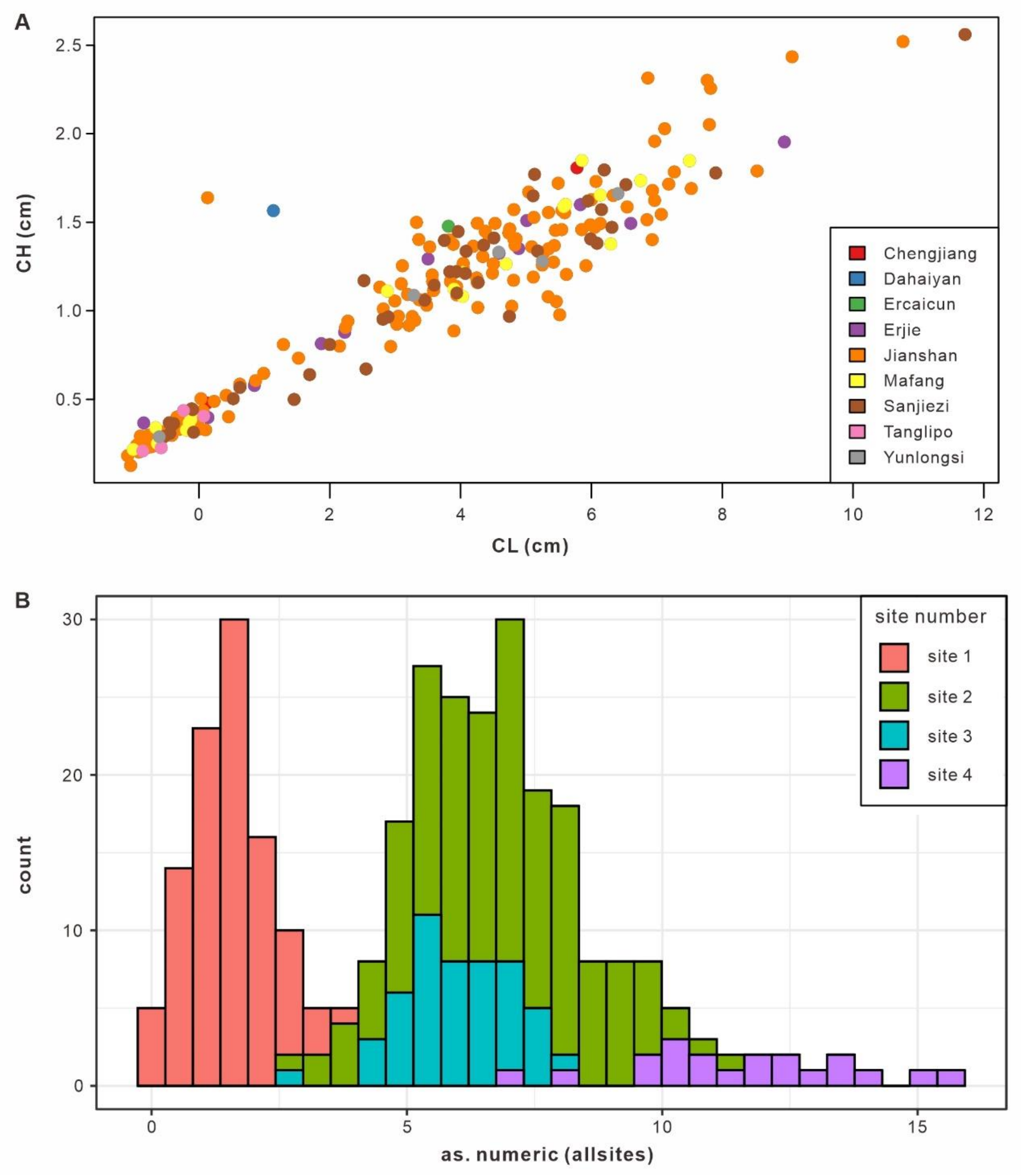

Supplementary Fig. S3. Scatter plots and histograms of CL and CH colored by different localities (A) with comparison to simulated data (B). Note how data from multiple sites is present across the sizes observed. It is possible to recover a multimodal distribution from sampling across multiple sites (B) however it is not expected to create the same type of distributions as observed in the empirical data.

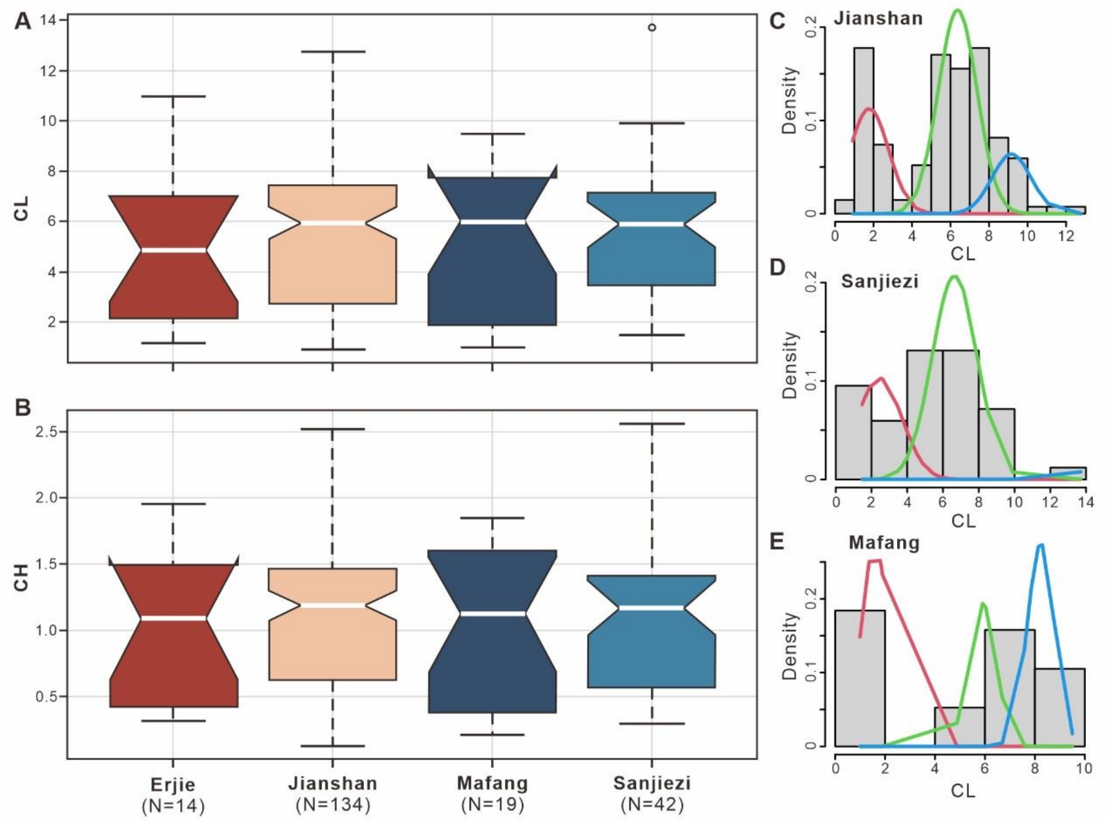

Supplementary Fig. S4. Specimen comparison of data across deposits with largest number of samples. Notched box and whisker plots of (A) claw length (CL) and (B) claw height (CH) of *Amplectobelua symbrachiata* frontal appendages from four sites with largest number of samples. Note how notches overlap in all four cases, and all show similar means, and interquartile ranges. Thus, the overlapping normal distributions seen are not likely a result of biostratinomic or taphonomic processes, and the data can be combined for further analysis. Source data provided in Supplementary Dataset 1. (C–E) show EM algorithm results for three distributions for CL data from three sites with largest sample sizes, Jianshan (C), Sanjiezi (D) and Mafang (E) respectively.

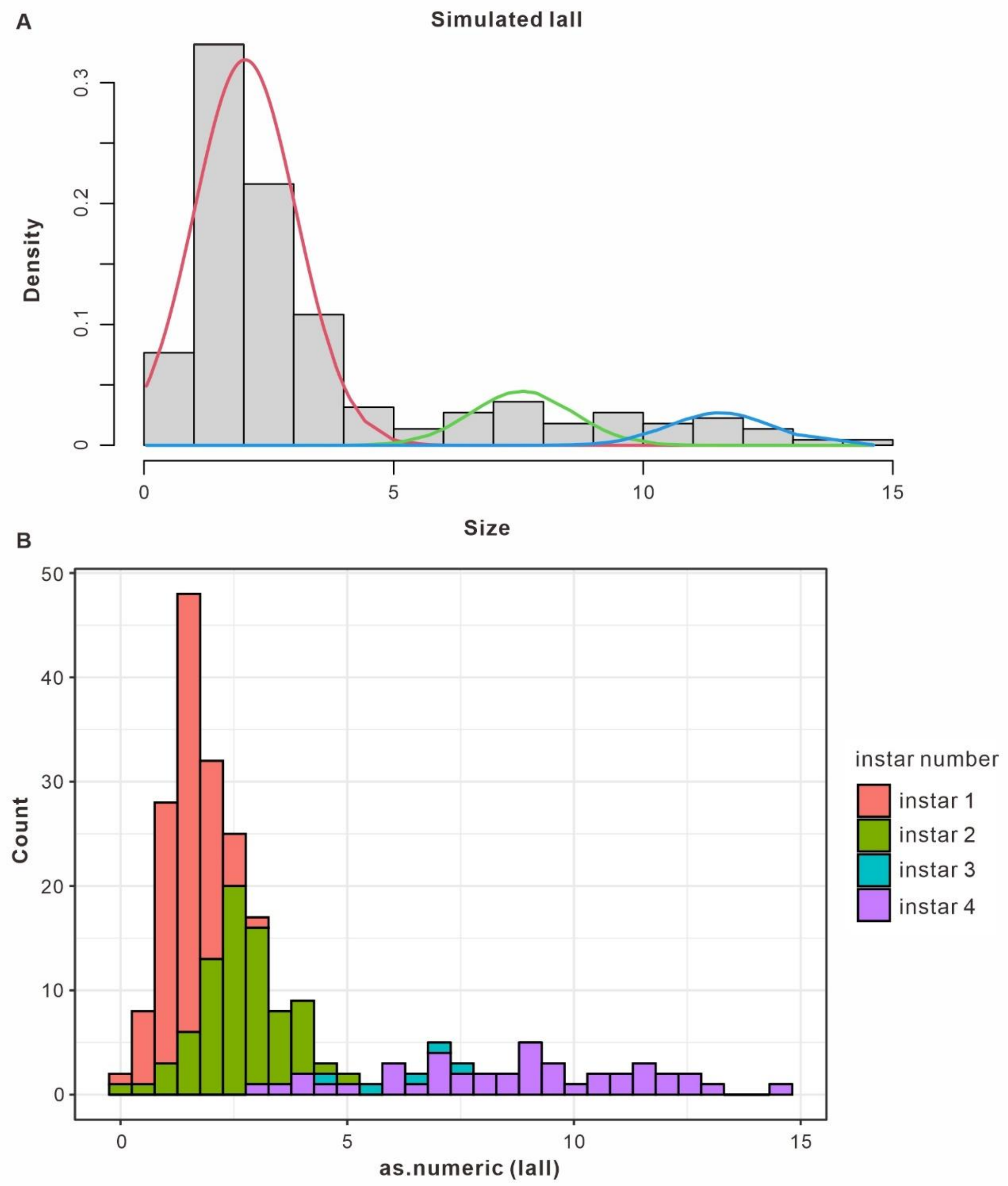

Supplementary Fig. S5. Analysis of simulated *Amplectobelua symbrachiata* instars. (A) Results of EM algorithm on simulated data from *InstarConstructor* function, showing how EM can recover three distributions from data with four original 'instars', if N is varied. (B) shows simulated data, colored by instar. Note that relative size of peaks and mean values are quite different from empirical data, but this example serves to show how it is plausible that a peak might be missing.

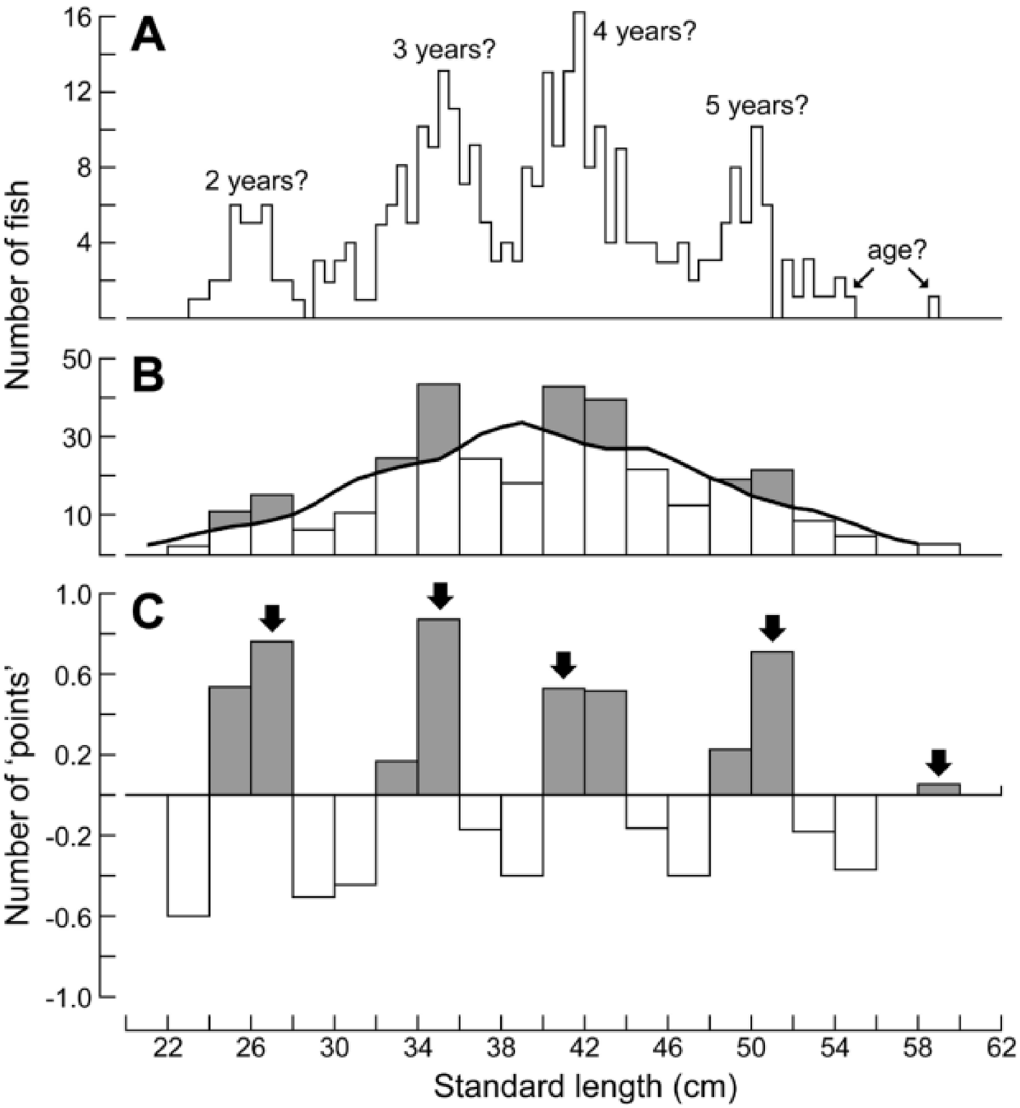

Supplementary Fig. S6. Length-frequency data on coral trout (*Plectropomus leopardus*) caught near Heron Island (Great Barrier Reef, Australia) in October 1971; from Ref. [94]). (A) Original data, with the peaks suggesting 'ages,' with question marks added; N = 319. Note the small class interval (0.5 cm), which is not appropriate for a fish reaching above 50 cm. (B) The same data, plotted in 2-cm class intervals, with mean frequencies (black line; incl. 5 classes) to identify peaks (in black, above running means) and intervening throughs. (C) the same data as in (B), after division of each. Note that well-structured peaks have been allotted similar numbers of points, irrespective of the number of individuals they represent. Arrows show the points used in the computation of ASP, or 'Available Sum of Peaks'; see also text (adapted from Ref. [95]).

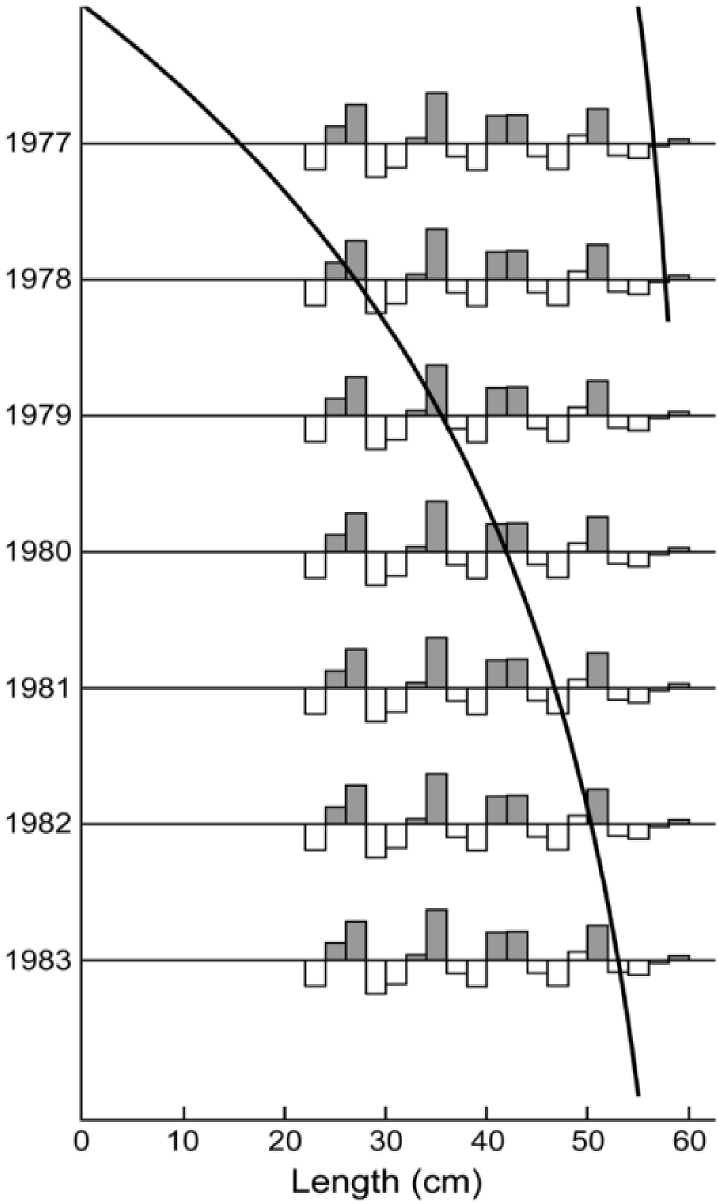

Supplementary Fig. S7. Growth of coral trout (*Plectropomus leopardus*) near Heron Island (Great Barrier Reef, Australia). Given the L/F data in Supplementary Fig. S6, and a value of L_∞ set at 65 cm, the scan for K estimated K= 0.29 yr^{-1} as best estimate, because the black peak hit (i.e., whose position is 'explained) by the VBGF generated as 'Explained Sum of Points' (ESP) that was a high fraction of the 'Available Sum of Points' (ASP, see Supplementary Fig. S6), i.e., $R_n = 10^{(EAP/ASP)}/10$ is maximized. The curve fitted by ELEFAN to the restructured L/F in Supplementary Fig. 7 is here repeated annually, under the assumption that growth patterns are the same for year to year; the years past 1977 are only for visualization. Note that the original assumption (by Ref. [94]) that the 4th peak from the left corresponds to an age of 5 years (see Supplementary Fig. S6A) is not tenable, given the VBGF parameters estimated here.

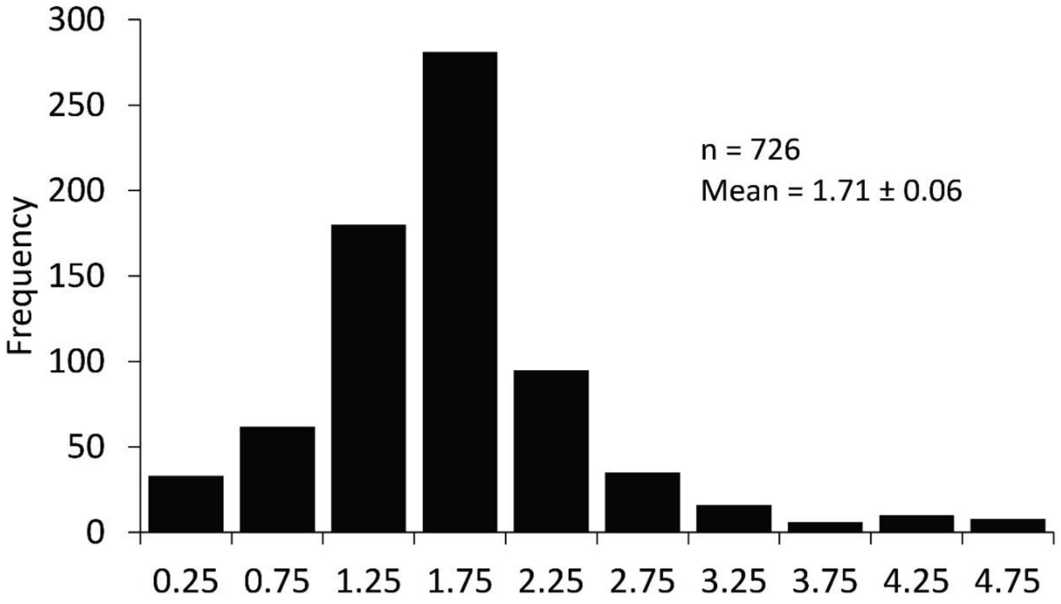

Supplementary Fig. S8. Frequency distribution of 726 estimates of the M/K ratio in SeaLifeBase (www.sealifebase.org), pertaining to 214 species in Recent non-fish water-breathing ectotherms in 72 families, 29 orders, 10 classes, and 5 phyla, i.e., Arthropoda (514 cases, 109 spp.), Chordata (8 cases, 5 spp.), Cnidaria (18 cases, 8 spp.), Echinodermata (15 cases, 10 spp.) and Mollusca (171 cases, 82 spp.).

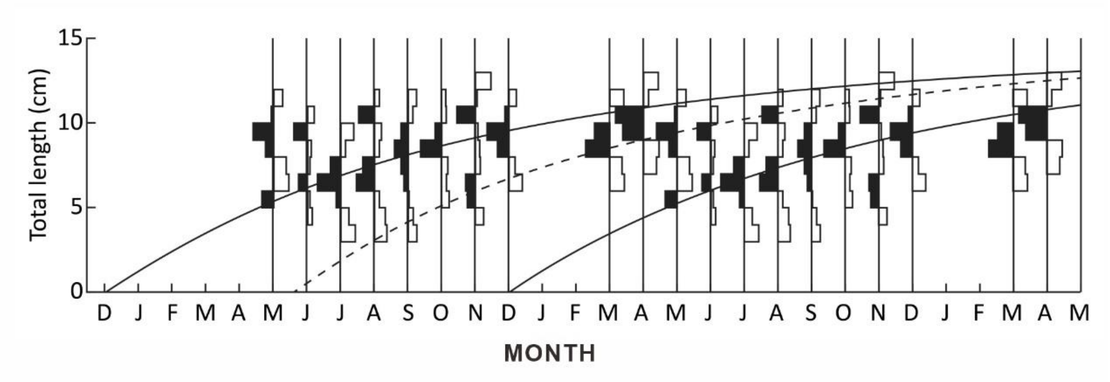

Supplementary Fig. S9. Growth curves for the ginger shrimp *Metapenaeus kutchensis* of Kandla (india). This based on 'restructured' length-frequency data in Ramamurthy [96] pertaining to the period from May 1959 to April 1960, and which are drawn twice to enable a better visualization of their trajectory. Note that the parameters (L_∞ and K) of the main growth curve (continuous lines) are estimated from the peaks (black, positive histograms) it 'hits' and the throughs (white, negative histograms) that it avoids, and is not influenced by the peaks that are ignored. Here, these peaks, presumably resulting from a second 'brood' or spawning event, are fitted with a secondary growth curve (dotted line) with parameters similar to those of the main growth curve. Important here is that the main growth curve and its parameter estimates are independent of whether spawning and the recruitment of young ginger shrimp to the adult population occur once or twice a year. The only effect that the presence of a secondary growth is that it reduces the goodness of fit of the main growth curve (modified from Pauly et al. [97]).

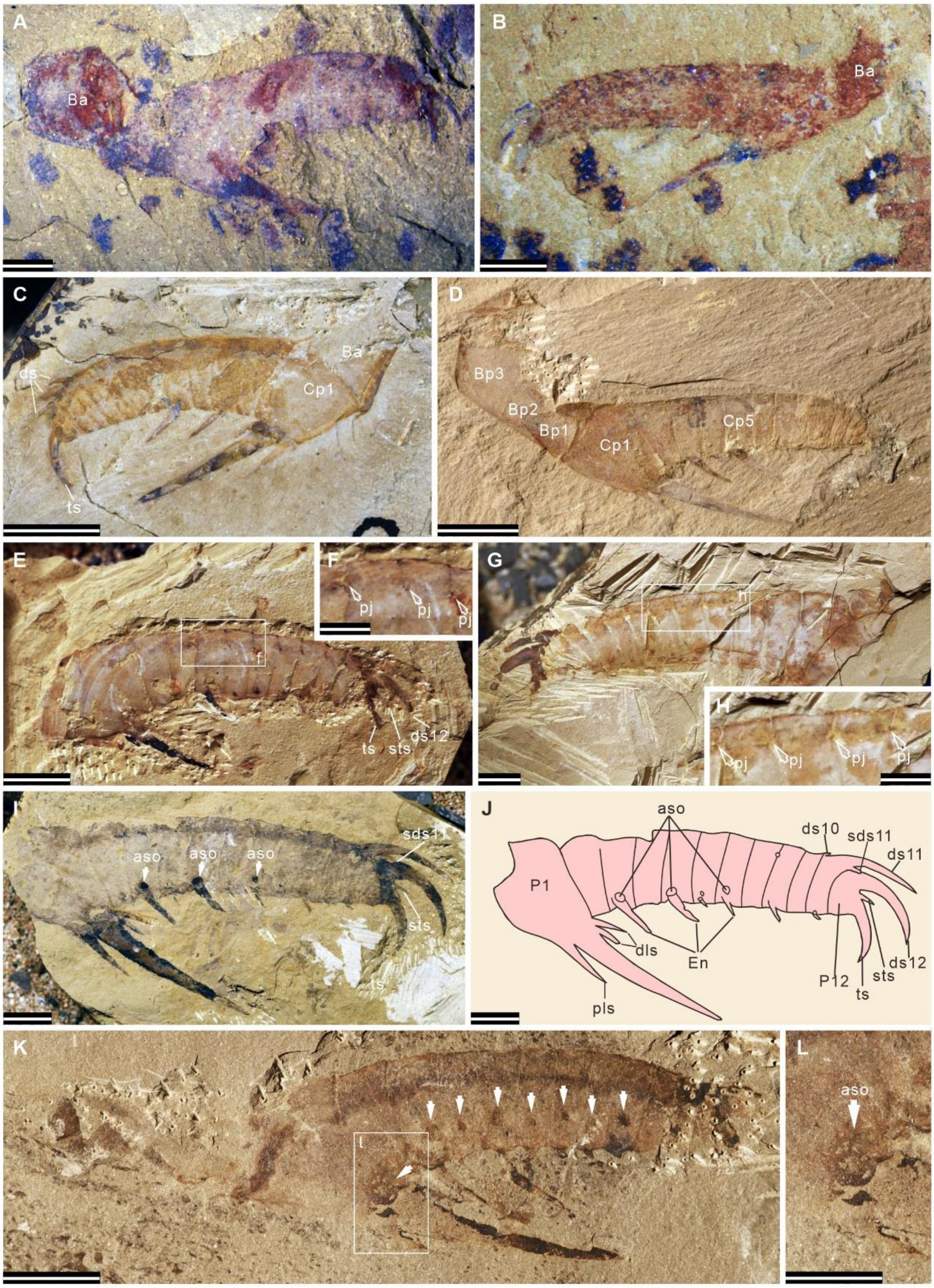

Supplementary Fig. S10. *Amplectobelua symbrachiata* appendages from the Cambrian (Stage 3) Chengjiang biota, including the smallest (B) and largest (K) specimens measured. (A) JS-0991, part. (B) JS-0850, the smallest juvenile appendage with poorly preserved base. (C) SJZ-139, juvenile appendage. (D) JS-0244, counterpart, the smallest complete appendage, showing the three base podomeres. (E) SJZ-171 appendage, showing the terminal spine and secondary terminal spine. (F) close-up of SJZ-171 (boxed in e), hollow arrows showing the pivot joint of podomeres. (G) SJZ-451 appendage. (H) close-up

of SJZ-451 (boxed in g), hollow arrows showing the pivot joint of podomeres. (I and J) SJZ-204, part, appendage with interpretative drawing (J), showing the secondary dorsal spine, secondary terminal spine and articulatory sockets (white solid arrows). (K) SJZ-642, part, nearly complete appendage, showing a series of enditic articulatory sockets (indicated by the solid white arrows). (L) close-up of basal part of En1 (boxed in K), showing the articulatory sockets of En1. Abbreviations: aso, articulatory socket; pe, peduncle; ds, dorsal spine; dls, distal lateral spine; pls, proximal lateral spine; pj, pivot joint; se, secondary endite; sds, secondary dorsal spine; sts, secondary terminal spine; ts, terminal spine. Scale bars, 10 mm (E, G, I, J), 5 mm (C, D, F, H, K, L), 2 mm (A, B).

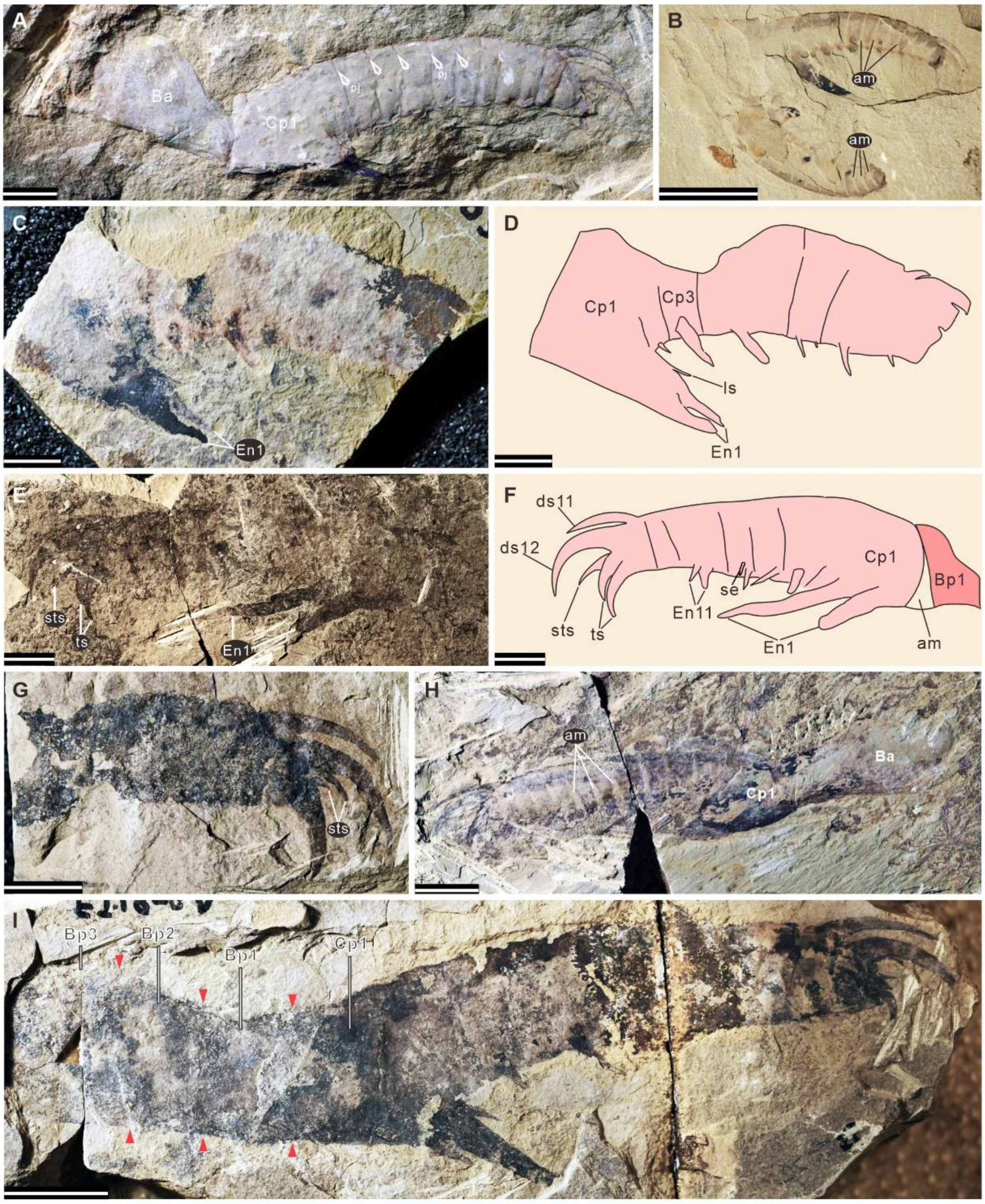

Supplementary Fig. S11. *Amplectobelua symbrachiata* frontal appendages from the Cambrian (Stage 3) Chengjiang biota. (A) SJZ-281, nearly complete appendage, hollow arrows showing the pivot joint of each podomeres (indicated by hollow arrows). (B) EJ-1889, part, paired complete appendages, showing the triangular arthrodial membrane between each podomeres. (C and D) JS-0075, part, appendage with interpretative drawing (D), showing the paired En1. (E and F) JS-0515, part, appendage with interpretative drawing (F), showing the paired En1 and secondary terminal spine on P12. (G) SJZ-144, part, showing the paired secondary terminal spine. (H) JS-0418, part, showing the triangular arthrodial membrane. (I) EJ-1908, the largest complete appendage, showing 12 claw podomeres and 3 base podomeres (red solid arrows indicate the boundaries between base podomeres). Abbreviations: am, arthrodial membrane; se, secondary endite. Other abbreviations as in Supplementary Fig. S10. Scale bars, 10 mm.

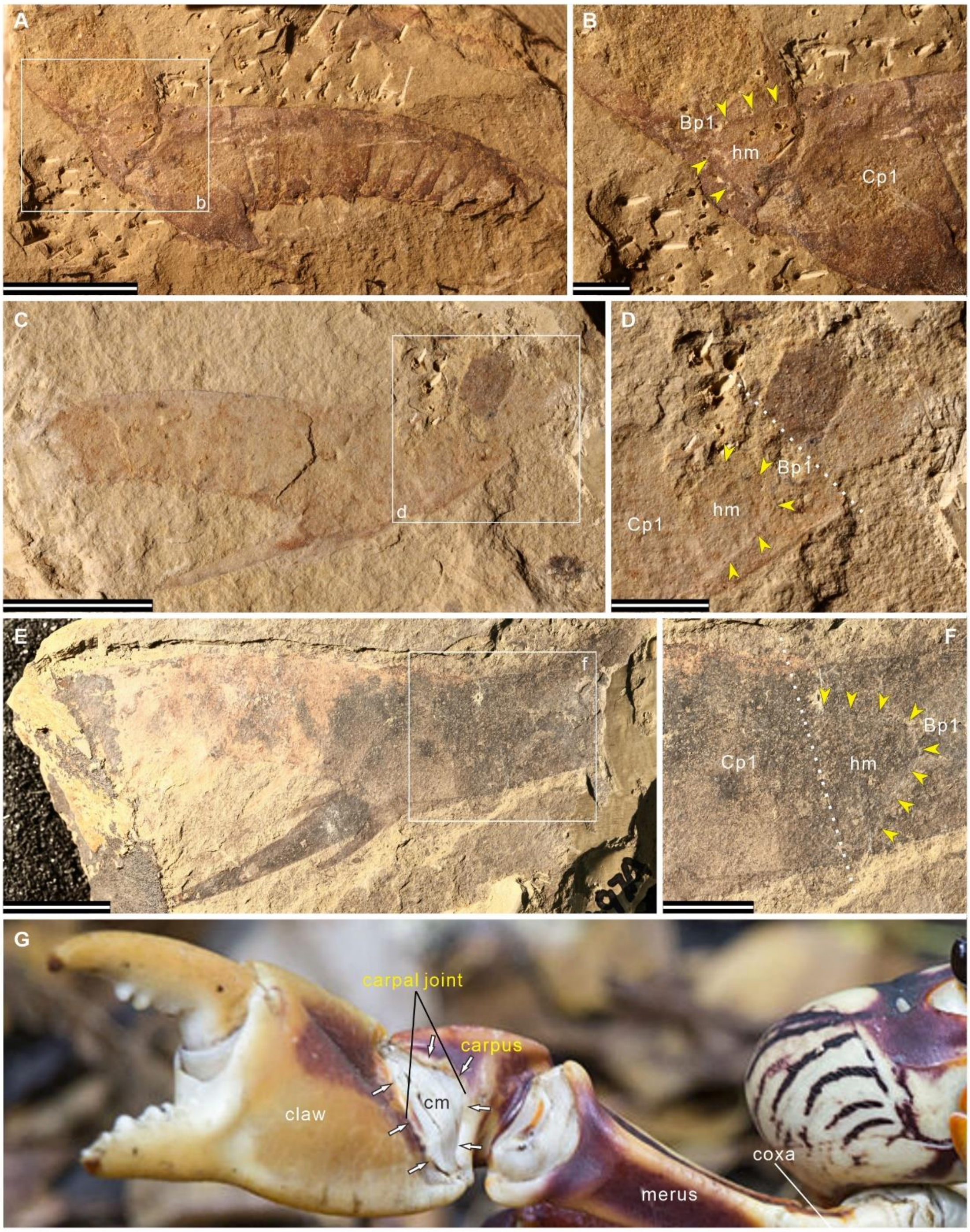

Supplementary Fig. S12. Comparison of *Amplectobelua symbrachiata* frontal appendages and chelipeds of extant crab. (A–F) Frontal appendages of *A. symbrachiata* in lateral-inner view. (A) SJZ-0324. (B) close-up of proximal region (white box b in A), yellow solid arrows indicating the hinge membrane on the distalmost base podomere. (C) EJ-1453. (D) close-up of proximal region (white box d in C), yellow solid arrows indicating the hinge membrane. (E) JS-0497. (F) close-up of proximal region (white box f in E), yellow solid arrows indicating the hinge membrane. (G) Cheliped of Cuban Red Crab, *Gecarcinus ruricola* (image courtesy of Wikipedia), white solid arrows indicating the triangular carpal membrane (no scale). Abbreviations: hm, hinge membrane; cm, carpal membrane. Scale bars, 5 mm (A, C, E), 2 mm (B, D, F).

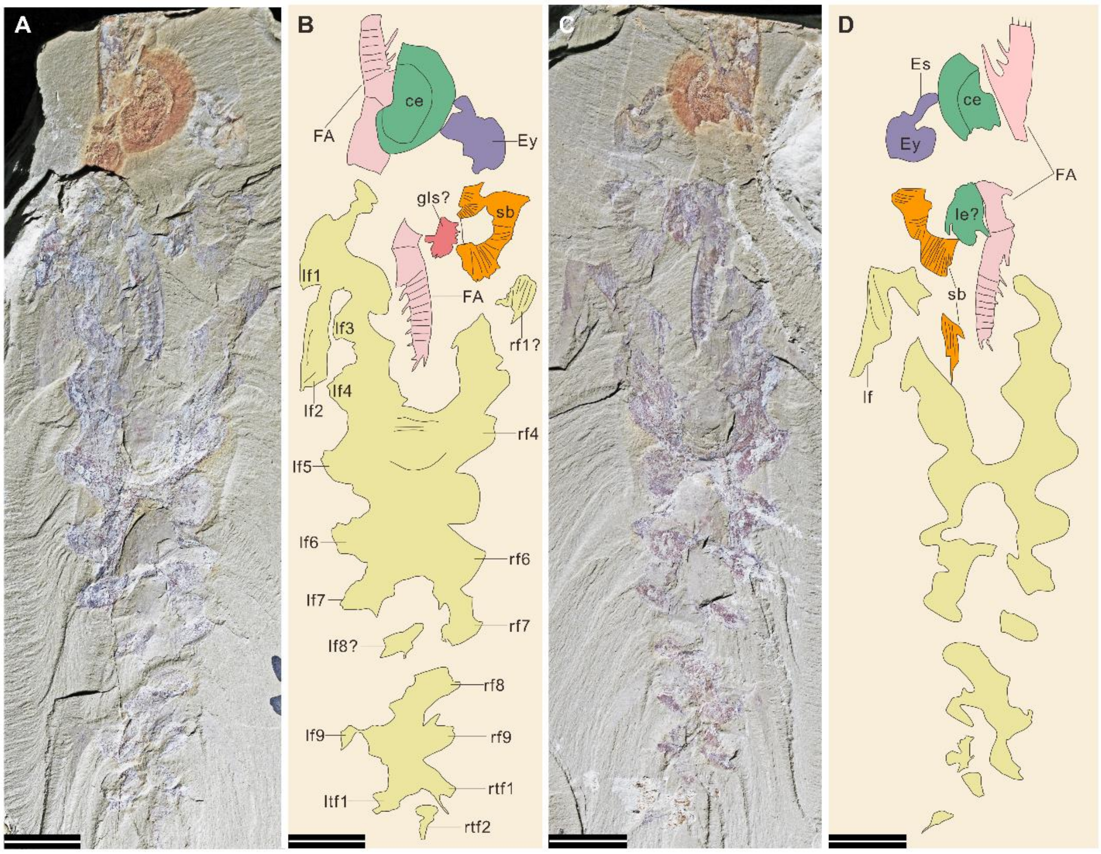

Supplementary Fig. S13. ***Amplectobelua symbrachiata*** **assemblage from the Cambrian (Stage 3) Chengjiang biota.** (A) Part of specimen JS-1917, general view of articulated assemblage, showing nine paired lateral body flaps, two tail fans, paired frontal appendages, and fragments of central element of head sclerite complex, eyes gnathobase-like structure and setal blades. (B) Interpretative drawing of (A). (C) Counterpart of specimen JS-1917, general view. (D) Interpretative drawing of (C). Abbreviations: ce, central element; es, eye stalk; ey, eye; gls, gnathobase-like structure; le, lateral element; lf, left body flap; ltf, left tail fan; Pe, P-element; rf, right body flap; rtf, right tail fan; sb, setal blades; tl, transverse line. Scale bars, 10 mm.

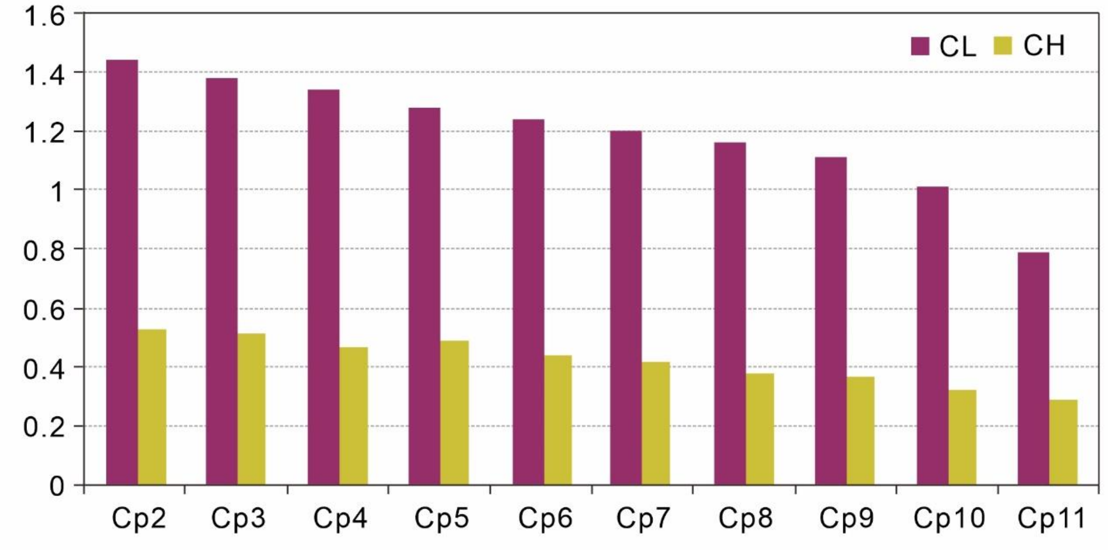

Supplementary Fig. S14. **Histogram showing the average lengths and heights of 10 appendage podomeres from Cp2 to Cp11.** This shows the slightly decrease in the length and height except for the length of Cp5, which is longer than Cp4. Source data provided in Supplementary Dataset 1.

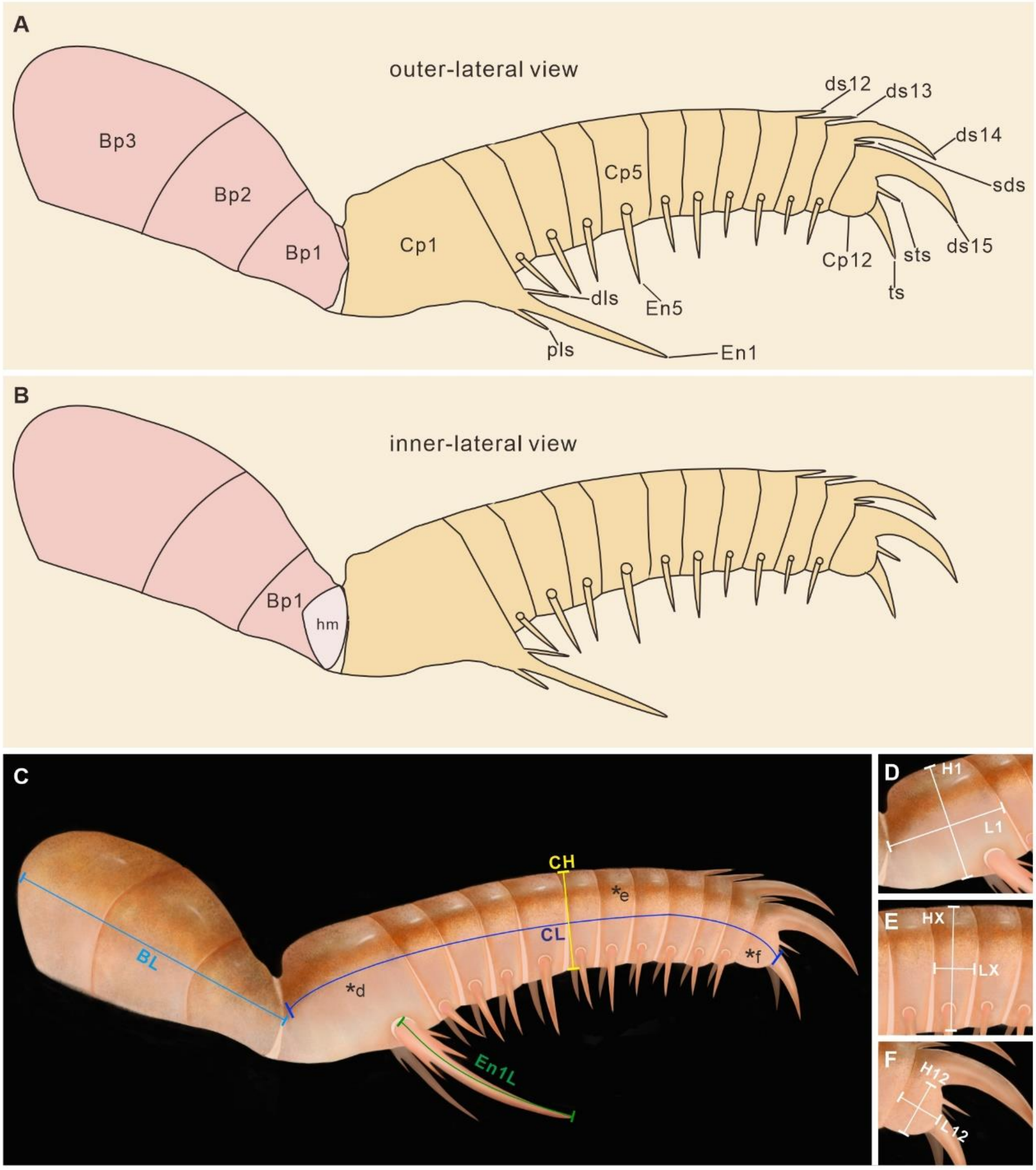

Supplementary Fig. S15. **Reconstruction of *Amplectobelua symbrachiata* frontal appendage and detailed explanations of measurements.** (A and B) Diagrammatic anatomical reconstructions of *Amplectobelua symbrachiata* frontal appendage based on this study. (A) Outer-lateral view. (B) Inner-lateral view, emphasizing the hinge membrane (hm). (C–F) Explanation of measurements taken for frontal appendage. (C) Claw length (CL), claw height (CH), base length (BL) and length of endite 1 (En1L). (D) Length and height of claw podomere 1 (L1, H1). (E) Length and height of claw podomere 2 to 11 (LX, LH). (F) Length and height of claw podomere 12 (L12, H12). Abbreviations as in Supplementary Figs. S10–S12.

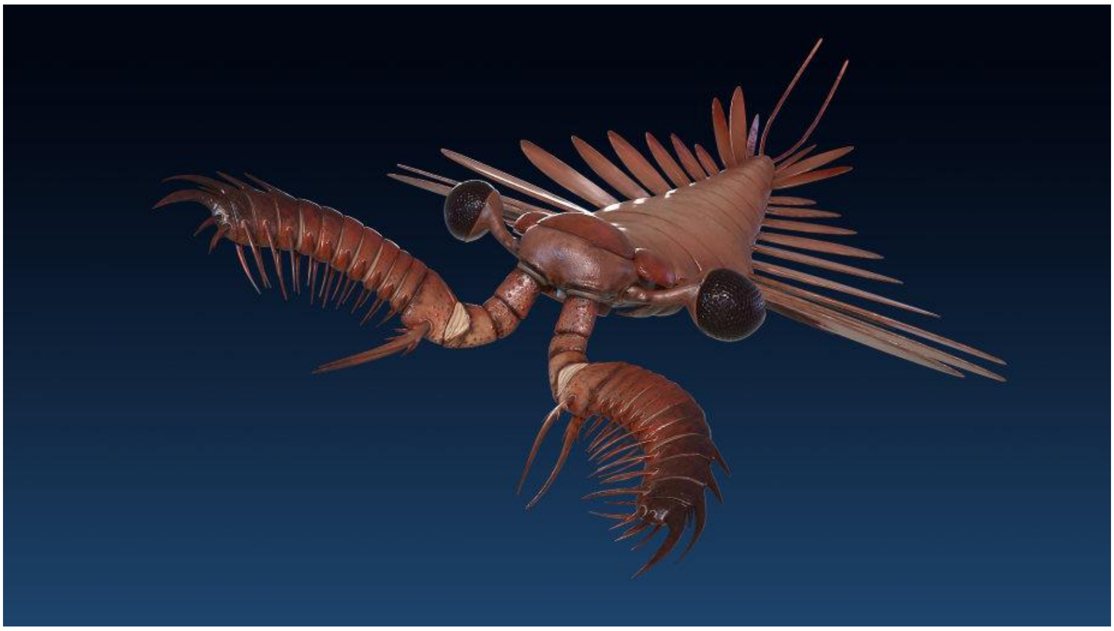

Supplementary Fig. S16. **Palaeoartistic reconstruction of *Amplectobelua symbrachiata*.** Drawn by Jingyu Liang (copyright by Northwest University, Xi'an, China; used with permission).

Supplementary Tables S1–S9

Supplementary Table S1. Basic statistics for variates measured (in cm) of frontal appendage in *Amplectobelua symbrachiata.*

Size class	Mean	Min	Max	Range	Variance	n
CL	5.35	0.91	13.71	12.8	7.77	224
CH	1.08	0.13	2.56	2.43	0.31	224
En1L	2.25	0.31	6.45	6.14	1.64	158
BL	2.55	1.1	4.44	3.33	1.62	6

Abbreviations: n, number of specimens for measurements. Dimensions CL, CH, En1L and BL represent claw length, claw height, En1 length, and base length, respectively. Source data provided in Supplementary Dataset 1.

Supplementary Table S2. Basic statistics for variates measured (in cm) of each distal articulated region podomere from Cp1 to Cp12. Source data provided in Dataset S1.

	Size class	Mean	Max.	Min.	Range	Variance	H/L ratio		n
Cp1	H	1.33	2.84	0.22	2.62	0.35	0.91		117
	L	1.46	3.36	0.22	3.14	0.48			
Cp2	H	1.44	3.29	0.23	3.06	0.42	2.81	Mean=2.92	260
	L	0.53	1.55	0.07	1.48	0.07			
Cp3	H	1.38	3.17	0.21	2.96	0.37	2.69		273
	L	0.52	1.22	0.08	1.14	0.06			
Cp4	H	1.34	2.92	0.19	2.73	0.33	2.90		298
	L	0.47	1.00	0.07	0.93	0.01			
Cp5	H	1.28	2.91	0.20	2.71	0.31	2.65		313
	L	0.49	1.44	0.07	1.37	0.05			
Cp6	H	1.24	2.80	0.18	2.62	0.31	2.89		310
	L	0.44	1.36	0.06	1.30	0.01			
Cp7	H	1.20	2.72	0.18	2.54	0.30	2.94		296
	L	0.42	1.33	0.05	1.28	0.04			
Cp8	H	1.16	2.68	0.17	2.51	0.28	3.14		282
	L	0.38	1.23	0.05	1.18	0.03			
Cp9	H	1.11	2.39	0.16	2.23	0.26	3.07		270
	L	0.37	1.11	0.05	1.06	0.03			
Cp10	H	1.01	2.18	0.14	2.04	0.24	3.25		238
	L	0.32	1.10	0.05	1.05	0.03			
Cp11	H	0.79	2.03	1.00	1.03	0.16	2.82		215

Cp12	L	0.29	0.77	0.04	0.73	0.02			189
	H	0.40	1.10	0.06	1.04	0.05	0.69		
	L	0.58	1.39	0.07	1.32	1.00			

Supplementary Table S3. Regression analyses of morphometrics data of *Amplectobelua symbrachiata*. Source data provided in Supplementary Dataset 1.

Independent variable (X)	dependent variable (Y)	Linear equation (lnY = alnX + b) (RMA)	r^2	95% confidence interval 'a'	n	T (b=1)	*p*	Growth
ln(CL)	ln(CH)	ln(CH) = 0.9817ln(CL) - 1.5669	0.9373	(0.95162, 1.0106)	224	57.613	<0.01	isometry
ln(CL)	ln(En1L)	ln(En1L) = 1.0308ln(CL) - 0.9084	0.9162	(0.98021, 1.0777)	160	41.287	<0.01	isometry
ln(L1)	ln(H1)	ln(H1) = 0.968ln(L1) - 0.0822	0.9398	(0.9199, 1.0208)	117	42.367	<0.01	isometry
ln(L2)	ln(H2)	ln(H2) = 0.9557ln(L2) + 0.9982	0.8921	(0.9148, 1.0013)	260	46.184	<0.01	isometry
ln(L3)	ln(H3)	ln(H3) = 0.9768ln(L3) + 0.9579	0.9271	(0.94078, 1.0125)	273	58.71	<0.01	isometry
ln(L4)	ln(H4)	ln(H4) = 0.9651ln(L4) + 1.0182	0.9265	(0.92803, 1.0001)	298	60.881	<0.01	isometry
ln(L5)	ln(H5)	ln(H5) = 0.9788ln(L5) + 0.9683	0.9314	(0.9512, 1.0053)	313	66.568	<0.01	isometry
ln(L6)	ln(H6)	ln(H6) = 0.9683ln(L6) + 1.0653	0.9279	(0.9358, 1.0013)	310	64.788	<0.01	isometry
ln(L7)	ln(H7)	ln(H7) = 0.973ln(L7) + 1.0871	0.9278	(0.9339, 1.0066)	296	64.06	<0.01	isometry
ln(L8)	ln(H8)	ln(H8) = 0.9714ln(L8) + 1.1199	0.9292	(0.9386, 1.0052)	282	60.633	<0.01	isometry
ln(L9)	ln(H9)	ln(H9) = 0.9825ln(L9) + 1.0863	0.9308	(0.9498, 1.0123)	270	59.818	<0.01	isometry
ln(L10)	ln(H10)	ln(H10) = 0.9743ln(L10) + 1.1256	0.9244	(0.93363, 1.0116)	238	53.499	<0.01	isometry
ln(L11)	ln(H11)	ln(H11) = 0.993ln(L11) + 1.0053	0.9072	(0.94551, 1.0374)	215	45.623	<0.01	isometry
ln(L12)	ln(H12)	ln(H12) = 1.0205ln(L12) - 0.3597	0.9257	(0.9768, 1.0624)	189	48.283	<0.01	isometry

Abbreviations: CL, claw length; CH, claw height; HX, height of podomere X; LX, length of podomere X; En1L, En1 length. RMA slope, 95% confidence intervals (CI) for RMA slope; n, Number of specimens used in particular analysis; r^2 = determination coefficient; T-t test.

Supplementary Table S4. Initial mean parameters input into EM algorithm.

	**Comp 1**	**Comp 2**	**Comp 3**	**Comp 4**
**DARH, 2 groups**	0.25	1.9		
**DARH, 3 groups**	0.25	1	2.2	
**DARH, 4 groups**	0.25	0.5	1	1.9
**DARL, 2 groups**	1.5	6.5		
**DARL, 3 groups**	1.5	6.5	13	
**DARL, 4 groups**	1.5	5	9	13

Two, three, and four overlapping normal distributions (Comp 1-4) were considered for both DARH and DARL data.

Supplementary Table S5. Results of the EM algorithms, showing final attributes lambda (mixing proportions) mu (mean parameters), and sigma (standard deviation) for each of the three normal distributions (Comp 1–3) recovered, for both CH and CL data.

**CH**			
	Comp 1	Comp 2	Comp 3
Lambda	0.328541	0.630427	0.0410319
Mu	0.406158	1.352825	2.2435094
Sigma	0.234846		
Log likelihood	-151.3261		
**CL**			
	Comp 1	Comp 2	Comp 3
Lambda	0.328534	0.589535	0.0819305
Mu	1.934319	6.628718	9.8025103
Sigma	1.148497		
Log likelihood	-514.9781		

Supplementary Table S6. Summary of CH and CL data for the four sites with largest number of specimens.

**CH**							
Site	Number of specimens	Min	1st quartile	Median	Mean	3rd quartile	Max
Erjie	14	0.3140	0.4567	1.0860	1.0212	1.4585	1.9540
Jianshan	135	0.1270	0.6265	1.1860	1.1049	1.4675	2.5210
Mafang	19	0.217	0.377	1.123	1.041	1.595	1.849
Sanjiezi	42	0.292	0.586	1.166	1.090	1.411	2.561
**CL**							
Site	Number of specimens	Min	1st quartile	Median	Mean	3rd quartile	Max
Erjie	14	1.156	2.315	4.865	5.052	6.980	10.950
Jianshan	135	0.910	2.745	5.941	5.510	7.441	12.763
Mafang	19	0.995	1.863	6.032	5.179	7.730	9.500
Sanjiezi	42	1.475	3.514	5.887	5.460	7.125	13.712

Units of size measurements: centimeters.

Supplementary Table S7. Results of EM algorithm for appendage length data from three sites with largest sample size compared to data from all sites (sample size in brackets). Ratio between first and second distribution means comparable for all three. Difference in mean for Comp 3 likely due to smaller sample sizes of large specimens.

	Comp 1 (mean)	Comp 2 (mean)	Comp 3 (mean)
All sites (224)	1.93	6.62	9.80
Jianshan (135)	1.78	6.37	9.16
Mafang (19)	1.59	5.91	8.22
Sanjiezi (42)	2.45	6.65	13.67

Supplementary Table S8. Growth ratio for CH and CL, between stages 2 and 1, and stages 3 and 2 respectively.

	**CH**	**CL**
Stage 2 from stage 1	3.33	3.43
Stage 3 from stage 2	1.66	1.48

Supplementary Table S9. Summaries of the EM algorithm as applied to simulated data using the *InstarConstructor* function, creating four instars with recruitment 100, 100, 10, 100, growth ratio 1.8, survival ratio 0.75, mean size at instar 1 1.5 and standard deviation of 0.5. Note that because this is data drawn randomly from a normal distribution, rerunning the code will result in slightly different results.

N[100,100,10,100]	Comp 1	Comp 2	Comp 3	Comp 4
**2 groups**				
Lambda	0.840712	0.159288		
Mu	2.141043	9.190913		
Sigma	1.298591			
Log likelihood	-466.5825			
**3 groups**				
Lambda	0.825094	0.107491	0.067415	
Mu	2.074697	7.367797	11.276542	
Sigma	1.018134			
Log likelihood	-439.8835			
**4 groups**				
Lambda	0.488039	0.337056	0.107493	0.0674126
Mu	2.074755	2.074755	7.367860	11.2766030
Sigma	1.0181367			
Log likelihood	-439.8835			

Supplementary References

1. Zhao FC, Zhu MY, Hu SX. Community structure and composition of the Cambrian Chengjiang biota. *Sci China Earth Sci* 2010; **53:**1784–1799.

2. Babcock LE and Zhang W. Stratigraphy, palaeontology, and depositional setting of the Chengjiang Lagerstätte (Lower Cambrian), Yunnan, China. *Palaeoworld* 2001; **13:** 66–86.

3. Hou XG, Siveter DJ and Siveter DJ *et al. The Cambrian fossils of Chengjiang, China: the flowering of early animal life*. Oxford: John Wiley & Sons, 2017.

4. Wu Y, Ma JX, Lin WL *et al*. New anomalocaridids (Panarthropoda, Radiodonta) from the lower Cambrian Chengjiang Lagerstätte, Biostratigraphic and paleobiogeographic implications. *Palaeogeogr Palaeoclimatol Palaeoecol* 2021; **569:** 110333.

5. Wu Y, Fu DJ and Ma JX *et al*. *Houcaris* gen. nov. from the early Cambrian (Stage 3) Chengjiang Lagerstätte expanded the palaeogeographical distribution of tamisiocaridids (Panarthropoda: Radiodonta). *Palz* 2021; **95:** 209–221.

6. Paterson JR, García-Bellido DC and Edgecombe GD. The early Cambrian Emu Bay Shale radiodonts revisited: morphology and systematics, *J Syst Palaeontol* 2023; **21:** 2225066.

7. Haug JT, Walosek D and Maas A *et al*. Functional morphology, ontogeny and evolution of mantis shrimp-like predators in the Cambrian. *Palaeontology* 2012; **55:** 369–99.

8. Cong PY, Daley AC and Edgecombe GD. *et al*. The functional head of the Cambrian radiodontan (stem-group Euarthropoda) *Amplectobelua symbrachiata*. *BMC Evol Biol* 2017; **17:** 208.

9. Lerosey-Aubril R and Pates S. New suspension-feeding radiodont suggests evolution of microplanktivory in Cambrian macronekton. *Nat Commun* 2018; **9:** 3774.

10. Guo J, Pates S and Cong PY *et al.* A new radiodont (stem Euarthropoda) frontal appendage with a mosaic of characters from the Cambrian (Series 2 Stage 3) Chengjiang biota. *Pap Palaeontol* 2019; **5:** 99–110.

11. Pates S, Daley AC and Butterfield NJ. First report of paired ventral endites in a hurdiid radiodont. *Zool Lett* 2019; **5:** 18.

12. Pates S, Daley AC and Edgecombe GD *et al.* Systematics, preservation, and biogeography of radiodonts from the southern Great Basin, USA, during the upper Dyeran (Cambrian Series 2, Stage 4). *Pap Palaeontol* 2021; **7:** 235–262.

13. Daley AC and Budd GE. New anomalocaridid appendages from the Burgess Shale, Canada. *Palaeontology* 2010; **53:** 721–738.

14. Cong PY, Edgecombe GD and Daley AC. *et al*. New radiodonts with gnathobase-like structures from the Cambrian Chengjiang biota and implications for the systematics of Radiodonta. *Pap Palaeontol* 2018; **4:** 605–621.

15. Van Roy P, Daley AC and Briggs DEG. Anomalocaridid trunk limb homology revealed by a giant filter-feeder with paired flaps. *Nature* 2015; **522:** 77–80.

16. C. T. Rueden *et al.*, ImageJ2: ImageJ for the next generation of scientific image data. *BMC Bioinformatics* 2017; **18:** 1–26.

17. Huxley JS. Relative growth and form transformation. *Proc R Soc London* 1950; **137:** 465–469.

18. Hammer DAT, Ryan PD and Hammer Ø *et al.* Past: Paleontological Statistics Software Package for Education and Data Analysis. *Palaeontol Electron* 2001; **4:** 178.

19. R Core Team. R Core Team. R: A language and environment for statistical computing. R Foundation for Statistical Computing, Vienna, Austria. http://www.r-project.org/(2020).

20. Benaglia T, Chauveau D and Hunter DR *et al.* mixtools: An R Package for Analyzing Mixture Models. *J Stat Softw* 2010; **32:** 1–29.

21. Flaherty L, Régnière J and Sweeney J. Number of instars and sexual dimorphism of *Tetropium fuscum* (Coleoptera: Cerambycidae) larvae determined by maximum likelihood. *Can Entomol* 2012; **144:** 720–6.

22. Sokal RR and Rohlf FJ. *Biometry*, 3d ed. New York: Freeman, 1995.

23. Sheldon PR. Trilobite size-frequency distributions, recognition of instars, and phyletic size changes. *Lethaia* 1988; **21:** 293–306.

24. Hunt G and Chapman RE. Evaluating hypotheses of instar-grouping in arthropods: a maximum likelihood approach. *Paleobiology* 2001; **27:** 466–84.

25. Chen C, Zhang CC and Li SY *et al*. Biological traits and life history of *Pagiophloeus tsushimanus* (Coleoptera: Curculionidae), a weevil pest on camphor trees in China. *J For Res* 2021; **32:** 1979–88.

26. Sedorko D, de Barros GEB and Netto RG *et al*. Multiple-Rusophycus assemblage from the Parnaíba Basin (NE Brazil) reflects trilobites as tracemakers and molting behavior. *Front Ecol Evol* 2023; **11:** 1117947.

27. Hunt G. Phenotypic variance inflation in fossil samples: an empirical assessment. *Paleobiology* 2004; **30:** 487–506.

28. Fu DJ, Zhang XL and Budd GE *et al*. Ontogeny and dimorphism of Isoxys auritus (Arthropoda) from the Early Cambrian Chengjiang biota, South China. *Gondwana Res* 2014; **25:** 975–82.

29. Wu Y, Fu DJ and Zhang XL *et al*. Dimorphism of Bivalvedarthropod *Branchiocaris*? *yunnanensis* from the early Cambrian Chengjiang biota, South China. *Acta Geol Sinica* 2016; **90:** 818–826.

30. Reyes-Maldonado R, Marie B and Ramírez A. Rearing methods and life cycle characteristics of *Chironomus* sp. Florida (Chironomidae: Diptera): A rapid-developing species for laboratory studies. *PloS ONE* 2021; **16:** e0247382.

31. Elmes GW, Thomas JA and Munguira ML *et al*. Larvae of lycaenid butterflies that parasitize ant colonies provide exceptions to normal insect growth rules. *Biol J Linn Soc* 2001; **73:** 259–278.

32. Velásquez N, Bautista K and Guevara M *et al.* Larval development and growth ratio in *Ischnura cruzi* de marmels, with description of last larval instar (Zygoptera: Coenagrionidae). *Odonatologica* 2009; **38:** 29–38.

33. Bhakat S. Deviation of Dyar's rule in the post-embryonic development in millipedes – a comprehensive analysis. [Preprint] (2020). https://doi.org/10.1101/2020.08.23.263848.

34. Causey NB. Studies on the life history and the ecology of the hothouse millipede, *Orthomorpha gracilis* (CL Koch 1847). *Am Midl Nat* 1943; **29:** 670–682.

35. Guimaraes FJ and Negreiros-Fransozo ML. Juvenile development and growth patterns in the mud crab *Eurytium limosum* (Say, 1818) (Decapoda, Brachyura, Xanthidae) under laboratory conditions. *J Nat Hist* 2005; **39:** 2145–61.

36. Minelli A and Fusco G. Arthropod Post-embryonic Development. In Minelli A, Boxshall G and Fusco G (eds.). *Arthropod Biology and Evolution. Molecules, Development, Morphology*, Berlin: Springer-Verlag, 2013, 91–122.

37. Yang XF, Kimmig J and Zhai DY *et al.* A juvenile-rich palaeocommunity of the lower Cambrian Chengjiang biota sheds light on palaeo-boom or palaeo-bust environments. *Nat. Ecol. Evol.* 2021; **5:** 1082–90.

38. Wolfe JM. Metamorphosis Is Ancestral for Crown Euarthropods, and Evolved in the Cambrian or Earlier. *Integr Comp Biol* 2017; **57:** 499–509.

39. Pauly D, Amarasinghe US and Chu E *et al*. The growth, respiration, and reproduction of crustaceans: a synthesis through the Gill- Oxygen Limitation Theory (GOLT). *J Crust Biol* 2022; **42:** 1–13

40. Pauly D and Holmes JD. Re-assessing growth and mortality estimates for the Ordovician trilobite *Triarthrus eatoni*. *Paleobiology* 2023; **49**, 120–30.

41. Pauly D. Beyond our original horizons: the tropicalization of Beverton and Holt. *Rev Fish Biol Fisher* 1998; **8:** 307–34.

42. Wetherall JA, Polovina JJ and Ralston S. Estimating growth and mortality in steady-state stocks from length-frequency data. In Pauly D and Morgan GR (eds.). *Length-based methods in fisheries research*, ICLARM Conference Proceedings 13, 1987, 53–74.

43. Gayanilo FC and Pauly D. FAO-ICLARM Stock Assessment Tools: Reference Manual. FAO Computerized Information Series/Fisheries. 1997, No. 8, Rome, 262 p.

44. Gayanilo FC Sparre P and Pauly D. FAO-ICLARM Stock Assessment Tools II (FiSAT II). Revised Version User's Guide. FAO Computerized Information Series (Fisheries), 2005, No. 8, Revised Version. FAO, Rome, 168 p.

45. Pauly D and Greenberg A. ELEFAN in R: A new tool for length-frequency analysis. *Fisheries Centre Research Reports* 2013; **21**: 52.

46. Mildenberger TK, Taylor MH and Wolff M. TropFishR: an R package for fisheries analysis with length-frequency data. *Methods Ecol Evol* 2017; **8:** 1520–7.

47. Palomares ML, Muck P and Mendo E *et al.* Growth of the *Peruvian anchoveta* (*Engraulis ringens*), 1953 to 1982. In: Pauly D and Tsukayama I (eds). *The Peruvian anchoveta and its upwelling ecosystem: three decades of change*. ICLARM Studies and Reviews 15, 1987, 117–41.

48. Ricker W. 1975. Computation and interpretation of biological statistics of fish populations. Bulletin of the Fisheries Research Board of Canada. Bulletin 191. Environment Canada, Ottawa, Canada. 382 p.

49. Longhurst A and Pauly D. *Ecology of Tropical Oceans.* (San Diego: Academic Press, 1987.

50. Pauly D. The Gill-Oxygen Limitation Theory (GOLT) and its critics. *Sci Adv* 2021; **7:** eabc6050.

51. Beverton RHJ and Holt SJ. A review of the lifespans and mortality rates of fish in nature and their relation to growth and other physiological characteristics. In Wolstenholme GEW and O'Connor M (eds.). *The Lifespan of Animals*. London: Churchill, 1959, 142-80.

52. Pauly D. On the interrelationships between natural mortality, growth parameters and mean environmental temperature in 175 fish stocks. *ICES J Mar Sci* 1980; **39:** 175–92.

53. Prince JD, Wilcox C and Hall N. How to estimate life history ratios to simplify data-poor fisheries assessment. *ICES J Mar Sci* **0,** 2023; 1–11 (2023).

54. Nielsen C. *Animal Evolution – Inter Relationships of the Living Phyla*. Oxford: Oxford University, 1995.

55. Collins D. The “evolution” of *Anomalocaris* and its classification in the arthropod class Dinocarida (nov.) and order Radiodonta (nov.). *J. Paleontol.* 1996; **70:** 280–93.

56. Hou XG, Bergström J and Ahlberg P. *Anomalocaris* and other large animals in the Lower Cambrian Chengjiang fauna of southwest China. *GFF* 1995; **117:** 163–83.

57. Cong PY, Ma XY and Hou XG *et al*. Brain structure resolves the segmental affinity of anomalocaridid appendages. *Nature* 2014; **513:** 538–42.

58. Zhang MJ, Wu Y, Lin WL *et al*. Amplectobeluid Radiodont *Guanshancaris* gen. nov. from the Lower Cambrian (Stage 4) Guanshan Lagerstätte of South China: Biostratigraphic and Paleobiogeographic Implications. *Biology* 2023; **12**: 583.

59. Wang YY, Huang DY and Hu SX. New anomalocardid frontal appendages from the Guanshan biota, eastern Yunnan. *Chin Sci Bull* 2013; **58:** 3937–42.

60. Moysiuk J and Caron J-B. Exceptional multifunctionality in the feeding apparatus of a mid-Cambrian radiodont. *Paleobiology* 2021; **47:** 704–24.

61. Pates S., Wolfe JM and Lerosey-Aubril R *et al*. New opabinid diversifies the weirdest wonders of the euarthropod stem group. *Proc R Soc B* 2022; **289:** 20212093.

62. Zeng H., Zhao FC and Zhu MY. *Innovatiocaris*, a complete radiodont from the early Cambrian Chengjiang Lagerstätte and its implications for the phylogeny of Radiodonta. *J Geol Soc* 2023; **180:** jgs2021–164.

63. Daley AC and Edgecombe GD. Morphology of *Anomalocaris canadensis* from the Burgess Shale. *J Paleontol* 2014; **88:** 68–91.

64. Jiao DG, Pates S and Lerosey-Aubril R *et al*. The endemic radiodionts of the Cambrian Stage 4 Guanshan Biota of South China. *Acta Palaeontol Pol* 2021; **66:** 255–74.

65. Moysiuk J and Caron J-B. A new hurdiid radiodont from the Burgess Shale evinces the exploitation of Cambrian infaunal food sources. *Proc R Soc B Biol Sci* 2019; **286:** 20191079.

66. Wu Y. Radiodont arthropods from the Cambrian Chengjiang Biota, South China. *Ph.D. Thesis*. Northwest University, Department of Geology, 2021.

67. Zeng H, Zhao FC and Yin ZJ*et al*. A new radiodontan oral cone with a unique combination of anatomical features from the early Cambrian Guanshan Lagerstätte, eastern Yunnan, South China. *J Paleontol* 2018; **92:** 40–8.

68. Whittington HB and Briggs DEG.The Largest Cambrian Animal, *Anomalocaris*, Burgess Shale, British Columbia. *Philos Trans R Soc B Biol Sci* **1985; 309:** 569–609.

69. Shu D, Chen L and Zhang XL *et al.* The Lower Cambrian KIN Fauna of Chengjiang Fossil Lagerstatte from Yunnan, China. *J Northwest Uni* 1992; **22**(suppl): 31–8 (in Chinese with English Summary).

70. Chen JY, Ramsköld L and Zhou GQ. Evidence for monophyly and arthropod affinity of Cambrian giant predators. *Science* **264:** 1304–8 (1994).

71. Chen JY, Zhou GQ and Zhu MY *et al*. *The Chengjiang biota: a unique window of the Cambrian explosion*. Taizhong: Naional Museum of Natural Science, 1996.

72. Chen JY and Zhou GQ. Biology of the Chengjiang Fauna. *Bull Nat Mus Natl Sci.* **10:** 11–105 (1997).

73. Hou XG, Bergström J and Wang H *et al*. *The Chengjiang Fauna -Exceptionally well-preserved animals from 530 million years ago.* Kunming: Yunnan Science and Techonology Press, 1999.

74. Luo HL, Hu SX and Chen L *et al*. *Early Cambrian Chengjiang Fauna from Kunming region, China*. Kunming: Yunnan Science and Technology Press, 1999.

75. Chen LZ, Luo HL and Hu SX *et al. Early Cambrian Chengjiang Fauna in eastern Yunnan China*. Kunming: Yunnan Science and Techonology Press, 2002.

76. Hou XG, Aldridge RJ and Bergstrom J *et al*. *The Cambrian fossils of Chengjiang, China - the flowering of early animal life.* Oxford: Blackwell Science, 2004.

77. Chen JY. *The dawn of the animal world*. Nanjing: Phoenix Science Press, 2004.

78. Zhao YL, Zhu MY and Babcock LE *et al.* Kaili Biota: a taphonomic window on diversification of metazoans from the basal Middle Cambrian: Guizhou, China. *Acta Geol Sin* 2005; **79:** 751–65.

79. Zeng H, Zhao FC and Yin ZJ *et al*. Morphology of diverse radiodontan head sclerites from the early Cambrian Chengjiang Lagerstätte, south-west China. *J Syst Palaeontol* 2017; **16:** 1–37.

80. Liu J, Lerosey-Aubril R and Steiner M *et al*. Origin of raptorial feeding in juvenile euarthropods revealed by a Cambrian radiodontan. *Natl Sci Rev* 2018; **5:** 863–9.

81. Pates S and Daley AC. The Kinzers Formation (Pennsylvania, USA): the most diverse assemblage of Cambrian Stage 4 radiodonts. *Geol Mag* 2019; **156:** 1233–46.

82. Briggs DEG. *Anomalocaris*, the largest known Cambrian arthropod. *Palaeontology* 1979; **22:** 631–64.

83. Lerosey-Aubril R, Kimmig J and Pates S *et al.* New exceptionally preserved panarthropods from the Drumian Wheeler Konservat-Lagerstätte of the House Range of Utah. *Pap Palaeontol* 2020; **6:** 501–31.

84. Bicknell R and Paterson J. Reappraising the early evidence of durophagy and drilling predation in the fossil record: Implications for escalation and the Cambrian Explosion. *Biol Rev* 2018; **93:** 693–708.

85. Usami Y, Kamono K and Kawamura K. How *Anomalocaris* swam in the Cambrian sea; a theoretical study based on hydrodynamics. In Sekimura T, Noji S and Ueno N et al. (eds.). *Morphogenesis and pattern formation in biological systems: experiments and models*. New York: Springer, 2003, 369–76.

86. Usami Y. Theoretical study on the body form and swimming pattern of *Anomalocaris* based on hydrodynamic simulation. *J Theor Biol* 2006; **238:** 11–7.

87. Paterson JR, Edgecombe GD and García-Bellido DC *et al.* Acute vision in the giant Cambrian predator *Anomalocaris* and the origin of compound eyes. *Nature* 2011; **480:** 237–40.

88. Izquierdo- López A and Caron JB. Extreme multisegmentation in a giant bivalved arthropod from the Cambrian Burgess Shale. *iScience* 2022; **25:** 104675.

89. Wehner R. Spatial vision in arthropods. In Autrum CH, Goodman J and Messenger B et al. (eds.). *Comparative Physiology and Evolution of Vision in Invertebrates*. Germany: Springer, 1981, 287–616.

90. Paterson JR, Edgecombe GD and García-Bellido DC. Disparate compound eyes of Cambrian radiodonts reveal their developmental growth mode and diverse visual ecology. *Sci Adv* 2020; **6:** eabc6721.

91. Liu Y, Lerosey-Aubril R and Audo D *et al.* Occurrence of the eudemersal radiodont Cambroraster in the early Cambrian Chengjiang Lagerstätte and the diversity of hurdiid ecomorphotypes. *Geol Mag* 2020; **157:** 1200–6.

92. Caron JB and Moysiuk J. A giant nektobenthic radiodont from the Burgess Shale and the significance of hurdiid carapace diversity. *R Soc Open Sci* **8:** 210664 (2021).

93. Yamada S. Ultrastructure and cuticle formation of the carapace in the myodocopan ostracod exemplified by Euphilomedes japonica (Crustacea: Ostracoda). *J Morphol* 2019; **280:** 809–26.

94. Goeden G. *A monograph of the coral trout*. Res. Bull. No. 1 Queensland Fish. Serv. Brisbane, 1978.

95. Pauly D and David N. ELEFAN I, a BASIC program for the objective extraction of growth parameters from length-frequency data. *Ber Dtsch Wiss Komm Meeresforsch* 1981; **28:** 205–11.

96. Ramamurthy S. Studies on the prawn fisheries of Kutch. In: Proceedings of the Symposium on Crustaceans held at Ernakulam, January 1965, Part IV. *Martine Biological Association of India*. India: Mandapam Camp, 1967, 1424–1436.

97. Paul D, Ingles J and Neal R. Application to shrimp stocks of objective methods for the estimation of growth, mortality and recruitment - related parameters from length-frequency data (ELELAN I and II). In: Gulland JA and Rothschild BI (eds.). *Penaeid shrimps-their biology and management*. England: Fishing News Books, 1984, 220–34.

Supplementary Dataset 1 (separate file)
Metrical data of frontal appendages of *Amplectobelua symbrachiata*.

Supplementary Dataset 2 (separate file)
CSV file referenced in Supplementary Dataset 6 and 7 containing the claw length and height data with locality.

Supplementary Dataset 3 (separate file)
CSV file referenced in Supplementary Dataset 7 containing the claw length and height data without locality.

Supplementary Dataset 4 (separate file)
Comparative growth data across other Euarthropoda documented in the literature.

Supplementary Dataset 5 (separate file)

CSV file referenced in Supplementary Dataset 8 containing comparative growth data across other Euarthropoda documented in the literature.

Supplementary Dataset 6 (separate file)
'Dataset S6_MixtureModel_Amplectobelua_ontogeny.R' is a R file containing the source code for Expectation-maximization (EM) algorithm used to determine the number of overlapping normal distributions.

Supplementary Dataset 7 (separate file)
'Dataset S7_PlottingGrowthData.R' is a R file containing the source code for determining how the growth ratios of *Amplectobelua symbrachiata* compare to other total-group euarthropods.

Supplementary Dataset 8 (separate file)
'Dataset S8_SimulateDistribution.R' is a R file containing the source code for determining how the growth ratios of *Amplectobelua symbrachiata* compare to other total-group euarthropods.
